# Hierarchical Dynamics in Polymer Nanocomposites—Insights from Neutron and X‑ray Scattering

**DOI:** 10.1021/acs.macromol.6c00919

**Published:** 2026-07-25

**Authors:** Tadanori Koga, Michihiro Nagao, Lutz Wiegart, Jan-Michael Y. Carrillo, Maya Endoh

**Affiliations:** † Department of Materials Science and Chemical Engineering, 12301 Stony Brook University, Stony Brook, New York 11794-2275, United States; ‡ Department of Chemistry, Stony Brook University, Stony Brook, New York 11794-3400, United States; § NIST Center for Neutron Research, 10833 National Institute of Standards and Technology, Gaithersburg, Maryland 20899-6102, United States; ∥ Department of Materials Science and Engineering, University of Maryland, College Park, Maryland 20742, United States; ⊥ National Synchrotron Light Source-II, 8099 Brookhaven National Laboratory, Upton, New York 11973, United States; # Center for Nanophase Materials Sciences, 6146 Oak Ridge National Laboratory, Oak Ridge, Tennessee 37831, United States

## Abstract

The incorporation of nanoparticles (NPs) into polymers is one of the most effective strategies for reinforcing polymeric materials. The impact of NP reinforcement is exemplified by automobile tires, where it enhances a broad range of performance metrics, including fracture toughness, wear resistance, wet traction, rolling resistance, and fatigue durability. Reinforcement arises from the coupled evolution of hierarchical structures and hierarchical dynamics of polymers and NPs across multiple length and time scales. A comprehensive understanding of reinforcement mechanisms remains elusive. The advancement of PNCs depends on fundamental research investigating the hierarchical structures and dynamics of polymers and NPs by employing integrated measurements, analysis, and predictions. In this Perspective, the emphasis is placed on elucidating the hierarchical dynamics of PNCs at the nano-to-mesoscale through the integration of neutron spin echo (NSE) and X-ray photon correlation spectroscopy (XPCS). We highlight recent advances in understanding the hierarchical dynamics of bound polymer, nanoparticle networks, and cross-linked polymer networks. NSE combined with isotope labeling provides unique access to the segmental and chain dynamics—including entanglement dynamics—of labeled polymer chains across length scales from subnanometers to tens of nanometers and time scales from picoseconds to several hundred nanoseconds. In contrast, XPCS provides unique access to the collective dynamics of hierarchical structures of NPs in a polymer matrix over relevant time scales (submilliseconds to 1000s of seconds) and length scales (nanometers to hundreds of nanometers). Furthermore, recent advancements in *in operando* XPCS have enabled the investigation of the nonequilibrium evolution of structures and dynamics in PNCs under industrially relevant processing conditions. Together with complementary computational and experimental approaches, these advanced neutron and X-ray scattering techniques establish quantitative hierarchical structure–dynamics–property relationships that provide a molecular foundation for the rational design of next-generation PNCs.

## Graphical abstract

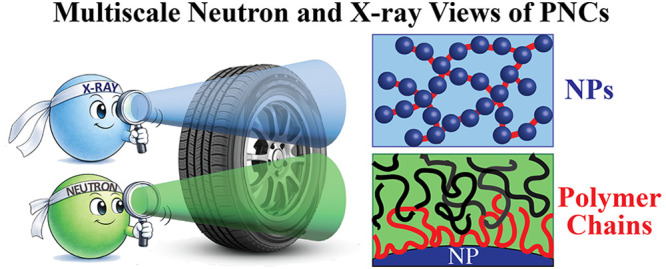

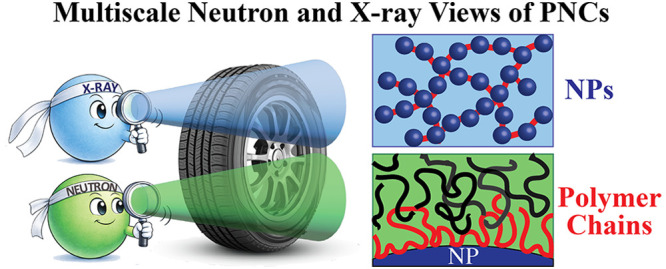

## 1 Introduction

Polymer nanocomposites (PNCs) continue to be of significant fundamental scientific and applied interest. This is due to their rich array of structural, dynamical, and mechanical behaviors, which involve both polymer and colloid science aspects.^1,2^ Carbon black (CB)-filled and silica (SiO_2_)-filled cross-linked elastomers are widely acknowledged as the most commercially successful PNCs.^3^ Specifically, automotive tires composed of CB-filled and SiO_2_-filled cross-linked elastomers represent a pivotal component of automotive supply chains. Given their exclusive role as the sole component of the vehicle that comes into contact with the road, tires wield a substantial influence on vehicle safety and fuel efficiency, irrespective of the choice of cars (gasoline vs electric vehicles). According to the Global Transport Scenarios 2050,^4^ the global total number of cars in 2050 will increase by about 2.5 times, in comparison with the year 2010. This underscores a significant demand for the advancement of reinforced synthetic elastomers with balanced, superior performance, encompassing durability and safety. This Perspective focuses on PNCs composed of spherical CB or SiO_2_ nanoparticles (NPs) dispersed in thermoplastics, elastomers, and thermosets, which serve as representative model systems and technologically important materials.

From an industrial perspective, reinforcement is inherently hierarchical, encompassing improvements in fracture toughness, wear resistance, wet traction, rolling resistance, and fatigue durability. The reinforcement of CB and SiO_2_-filled PNCs is increasingly recognized as a multiscale phenomenon governed by the intricate interplay among NP morphology, surface chemistry, polymer-NP interactions, and polymer chain dynamics,^5–12^ as CB and SiO_2_ NPs not only serve as rigid reinforcing phases but also modify polymer conformations, segmental mobility, entanglement dynamics, and stress relaxation within the NP-polymer interfacial regions. These molecular- and mesoscale modifications ultimately govern the reinforcement effect.

As illustrated in Figure 1, the structures and dynamics of polymers and NPs span an exceptionally broad range of length scales, extending from angstroms to micrometers, and time scales, ranging from femtoseconds to seconds. Establishing quantitative structure–property relationships has long been a central objective in PNC research for the past few decades.^13^ However, more recent studies have demonstrated that the structural descriptors alone are insufficient to explain the macroscopic behavior, processability, and long-term performance of PNCs.^2,14–17^ Because reinforcement arises from the coupled evolution of hierarchical structures and dynamics of polymers and NPs across multiple length and time scales, the molecular underpinnings of reinforcement in PNCs remain a subject of debate in this comparatively mature field.^1^ Consequently, the rational design of advanced PNCs with balanced performance, durability, and manufacturability requires a comprehensive understanding of the interconnected nature of hierarchical structures and dynamics.

**Figure 1:**
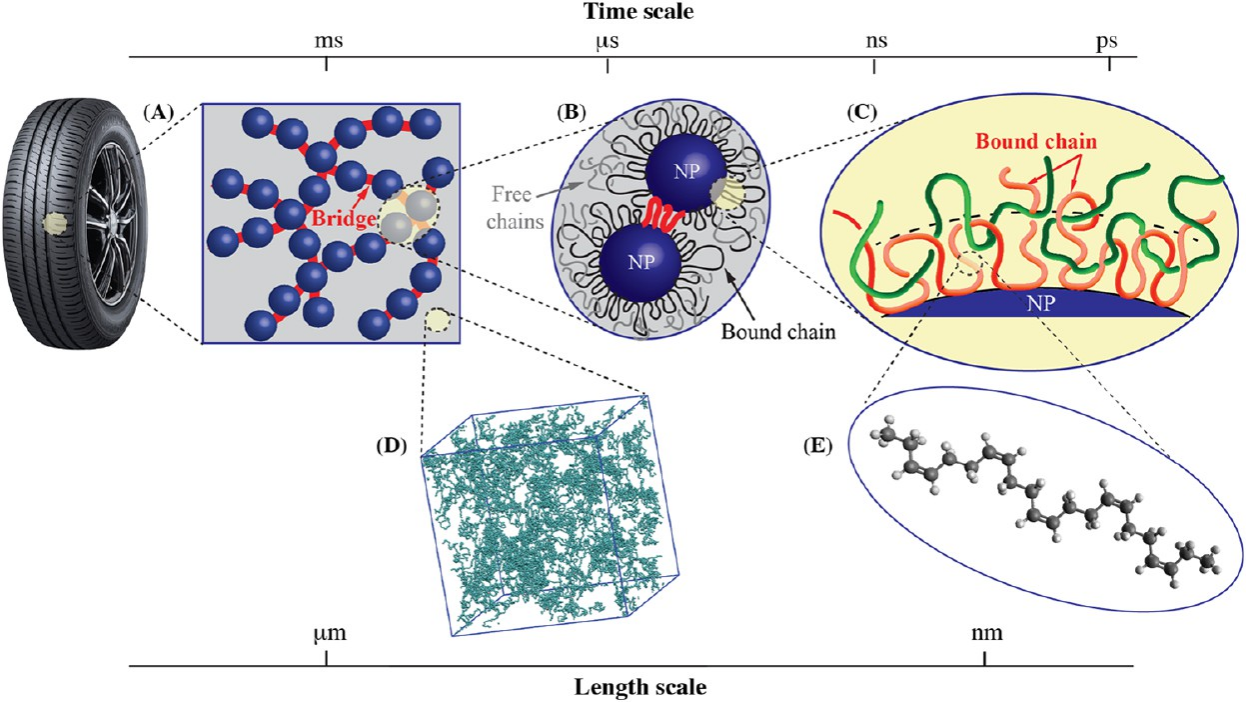
Schematic illustrations of the relevant hierarchical structures and dynamics that are responsible for the macroscopic properties of PNCs. (A) The formation of NP network structures via polymer-mediated bridges between neighboring NPs. (B) The BP and polymer bridges (highlighted in red) on the NP surface. (C) Topological structures of BP which are composed of three segment sequences. “Trains” are defined as adsorbed segments, “loops” are defined as sequences of free segments that connect successive trains), and “tails” are defined as nonadsorbed chain ends. (D) Polymer network structures. (E) Polymer segments.

This Perspective is organized around three hierarchical dynamic entities that govern reinforcement in PNCs: (i) bound polymer (BP), (ii) NP networks, and (iii) polymer networks (see Figure 1), as revealed by X-ray photon correlation spectroscopy (XPCS) and neutron spin echo (NSE). Comprehensive reviews of polymers and NP dynamics in PNCs are available elsewhere.^2,15^ Although the impact of NP morphology (aggregated NPs) on reinforcement has been a subject of considerable interest,^9,10^ we focus exclusively on the utilization of spherical NPs in the context of advanced scattering measurements, analysis, and predictions. Furthermore, the integration of neutron backscattering (NBS) is emphasized, as it extends the investigation of length and time scales. The spatial and temporal coverages of these spectrometers are summarized in Figure 2. Coarse-grained molecular dynamics (CGMD) simulations^18,19^ are also employed as complementary tools, as they provide access to dynamical details that are not accessible through experimental methods. Together with structural information obtained from small-angle X-ray scattering (SAXS) and small-angle neutron scattering (SANS), these techniques establish hierarchical correlations between the microscopic feature, molecular dynamics, and the macroscopic properties. Despite the complementary capabilities of NSE/NBS and XPCS, gaps remain in the experimentally accessible length and time scales (see Figure 2). Bridging these gaps requires additional techniques, including broadband dielectric spectroscopy (BDS)^17,20^ and nuclear magnetic resonance (NMR) spectroscopy,^21,22^ which provide complementary dynamic information over time and length scales inaccessible to scattering methods alone.

**Figure 2:**
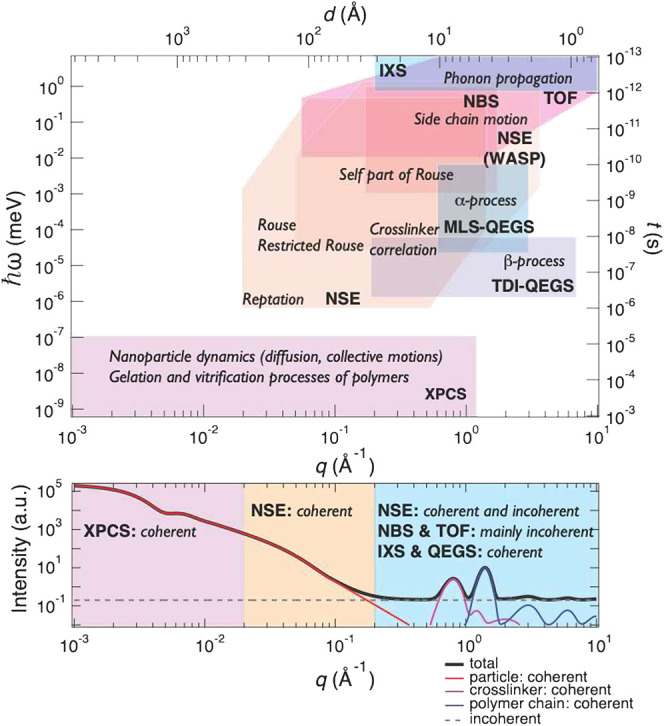
Top panel: Schematic representation of the approximate dynamic ranges of various spectroscopic techniques employing quantum beams for PNCs. A concise summary of the techniques’ respective acronyms is provided in Abbreviations and Acronyms. Typical dynamics processes in PNCs are also provided. Bottom panel: A model static structure factor of a PNC system and the typical methods for utilizing different types of X-ray and neutron spectroscopy are emphasized, alongside coherent and incoherent scattering components. Scattering from NP appears at low *q* as a coherent scattering signal, where XPCS can be used to measure NP dynamics. In the event that the scattering from structural heterogeneity, such as polymerization and gelation, may emerge, and XPCS can probe the corresponding dynamics as well (see Section 5). As *q* increases, the NP scattering intensity decreases, and surface polymer scattering emerges; here, NSE spectroscopy is a useful tool for measuring coherent polymer chain dynamics. Depending on the deuterium labeling, NSE is also valuable for probing chain dynamics in the bulk. Once the nanoparticle scattering fades, neutron incoherent scattering dominates. In such circumstances, the self-dynamics of hydrogen are emphasized when NBS and/or TOF spectrometers are employed. Generally, such incoherent scattering does not appear in X-ray scattering techniques. At specific *q* values, local structural correlations—such as cross-linker and polymer chain correlations—contribute to the static structure factor, thereby enabling coherent dynamics to be measured using various spectroscopic techniques. Notably, deuteration is required for neutrons to resolve these coherent dynamics, whereas partial deuteration can tune the coherent signal intensity, thereby enabling researchers to isolate specific dynamic components.

The BP is composed of polymer chains that physically adsorb on the surfaces of NP, as it has been observed in various PNCs.^23–46^ The thickness of the BP is typically commensurate with the radius of gyration (*R*_g_) of adsorbed polymer chains (see Figure 1B and C). It is noteworthy that the BP corresponds to the so-called “bound rubber” in the rubber industry, which physically or chemically adsorbs on the CB or SiO_2_ surface.^47–49^ To avoid any potential for confusion, the term bound polymer or BP will be used to denote bound chains on the NP surface consistently throughout this Perspective. In the context of commercial tire production, where a high NP volume fraction is a critical aspect, the interparticle separation distance between neighboring NPs decreases, and chain confinement induced by neighboring NPs emerges.^50,51^ At large enough interfacial polymer-NP attraction, the discrete BP becomes thermodynamically disfavored, and relatively *tight* polymer-mediated “bridges” ^52–54^ between neighboring NPs emerge, leading to a network-like microstructure^52^ that reinforces PNCs as a large scale skeleton.^10^ This NP network structure is the primary factor contributing to mechanical property enhancement (Figure 1A).^9,38,43,52,55–61^ A polymer network is defined as a three-dimensional cross-linked polymer network, a process often designated as curing^62^ (see Figure 1D). Because curing continuously modifies both the network topology and the underlying molecular dynamics, cross-linked thermosets provide an ideal model system for investigating hierarchical dynamic arrest across multiple length and time scales. Moreover, unlike BP and NP networks, whose hierarchical dynamics evolve within pre-existing structures, thermosets provide an opportunity to directly observe the emergence of hierarchical dynamics during network formation itself.

We first provide a concise overview of the NSE/NBS, CGMD, and XPCS methodologies for investigating the hierarchical structures and dynamics of PNCs. We then highlight several recent advancements that have substantially improved our understanding of the dynamic behavior of three key hierarchical constituents governing reinforcement: BP, NP networks, and cross-linked polymer networks. For BP, neutron scattering, combined with isotope labeling and CGMD simulations, enables direct characterization of interfacial polymer structure on nanometer length scales together with their dynamics on time scales extending to several hundred nanoseconds in model CB and SiO_2_-filled elastomers. For NP network, XPCS, in conjunction with SAXS, provides simultaneous access to network structure spanning hundreds of nanometers and their collective dynamics over an exceptionally broad temporal window (10^–3^ s < *t* < 10^3^ s). As a representative model system, we focus on spherical SiO_2_ NPs dispersed in attractive poly­(2-vinylpyridine) (P2VP) melts, which constitute a prototypical example of globally homogeneous, “well dispersed” PNCs.^33,41,63^ Finally, we discuss the emerging opportunities afforded by *in operando* XPCS measurements for probing the nonequilibrium evolution of the structures and dynamics of three-dimensional cross-linked polymer networks in NP-filled thermosets under relevant industrial processing conditions. Collectively, these complementary methodologies establish a multiscale framework for elucidating the coupled evolution of BP, NP networks, and polymer networks across the length and time scales governing reinforcement in PNCs. Finally, we discuss future opportunities at the intersection of advanced scattering methods, MD simulations, and modern machine-learning methodologies for polymer science and engineering, which will accelerate the molecular design of next-generation PNC materials. We hope that this Perspective will serve as a useful guideline for researchers seeking to measure, analyze, and elucidate the underlying connections among the structures, dynamics, processing, and properties in complex macromolecular systems.

## 2 Principles of Experimentation and Computation

### 2.1 Quasielastic Neutron Scattering (QENS)

In the context of scattering experiments, the structural characteristics and dynamic properties of materials are investigated by the measurement of the intensity of the outgoing wave relative to the incident wave.^64^ The dynamic range and the target system of a scattering experiment depend on the wavelength, energy, and nature of interactions of the probe. Visible light possesses a wavelength of several hundred nm, a property that renders it well-suited for the study of relatively large structures. In contrast, X-rays and neutrons have wavelengths on the order of sub nm, rendering them optimal for the study of atomic to molecular-scale structures. X-rays, with a wavelength of 0.1 nm, possess energies on the order of 10 keV, in comparison, neutrons, with a similar wavelength, possess energies on the order of 10 meV, which is comparable to the thermal energy. Therefore, neutrons are particularly well-suited to probing thermal fluctuations in materials.

In this section, we briefly introduce the features of QENS. Readers are encouraged to refer to textbooks and review articles^65–70^ for more in-depth information. Given that neutrons interact with materials via neutron-nucleus interactions (with magnetic interactions being negligible for PNCs), the neutron scattering length varies from isotope to isotope, exhibiting no systematic relation to atomic number. To illustrate, the substitution of hydrogen (H) atoms with deuterium (D) results in a substantial alternation of the neutron scattering length, while maintaining the chemical nature of the molecules relatively unaltered. This method enables the selective observation of the structure and dynamics of materials in a targeted manner by accentuating specific regions of the sample through the use of deuteration. The scattering length also depends on the nuclear spin state. Given the random distribution of atoms with different nuclear spins within the system, the resulting scattering, which is contingent on the nuclear spin state, manifests as incoherent scattering. In contrast to the coherent scattering, which gives rise to interference patterns, incoherent scattering depends on the number of atoms within the system and shows uniform intensity in all directions. The incoherent scattering cross section of H is considerably larger than the scattering cross sections of other nuclei. Consequently, the incoherent scattering from H has been a focal point in several neutron scattering experiments, particularly those involving time-of-flight (TOF) QENS or NBS.

The incident neutron beam, characterized by a defined wavenumber, ***k***_i_, and energy, *E*_i_, is directed toward the sample. The neutron-nuclei interactions may result in changes in the final wavenumber, ***k***_f_, and energy, *E*_f_. During the scattering process, the momentum and energy of the system are conserved. The momentum transfer is defined as *ℏ* ***q*** = *ℏ* ***k***_*f*_ – *ℏ* ***k***_*i*_, and the energy transfer is defined as *E* = *ℏω* = *E*_*f*_ – *E*_*i*_. In these equations, *h* = 2*πℏ* is the Planck constant, ***q*** = ***k***_*f*_ – ***k***_*i*_ is the wavevector transfer, and ω is the frequency difference between the incident and final neutrons, respectively. It is important to note that, while ***q*** is a vectorial parameter, for isotropic samples, only the magnitude of the wavevector transfer is considered, given by | ***q*** | = *q*. The probability of scattering is expressed through the double differential scattering cross section $\frac{d^{2}\sigma }{d\Omega d\omega }$ , which indicates the probability of neutron scattering in a solid angle between Ω and Ω + *d*Ω. This probability is contingent upon energy transfer between *ℏω* and *ℏω* + *ℏdω*, and is related to the dynamic structure factor *S*(*q*,ω) = ***ℏ*** *S*(*q*,*E*). Both coherent and incoherent scattering contribute to *S*(*q*,ω) as a linear combination of each component, *S*(*q*,ω) = *S*_coh_(*q*,ω) + *S*_inc_(*q*,ω). The dynamic structure factor is defined as the time Fourier transform of the intermediate scattering function as follows:

1 $$S_{\mathrm{coh}}\left(q,\omega \right)=\frac{1}{2\pi }\int \mathrm{dt}e^{i\omega t}S_{\mathrm{coh}}\left(q,t\right)$$

2 $$S_{\mathrm{inc}}\left(q,\omega \right)=\frac{1}{2\pi }\int \mathrm{dt}e^{i\omega t}S_{\mathrm{inc}}\left(q,t\right)$$

where *S*_coh_(*q*,*t*) and *S*_inc_(*q*,*t*) represent the coherent and incoherent intermediate scattering functions, respectively. These functions are expressed as follows:

3 $$S_{\mathrm{coh}}\left(q,t\right)=\frac{1}{N}\left\langle \sum_{i,j}b_{i}b_{j}e^{\mathrm{iq}\left[r_{i}\left(t\right)-r_{j}\left(0\right)\right]}\right\rangle$$

4 $$S_{\mathrm{inc}}\left(q,t\right)=\frac{1}{4\pi N}\left\langle \sum_{i}\sigma_{i}e^{\mathrm{iq}\left[r_{i}\left(t\right)-r_{i}\left(0\right)\right]}\right\rangle$$

with *N* denoting the number of atoms in the system, *b_i_* and *b_j_* representing the scattering lengths of the *i*-th and *j*-th atoms, respectively, σ_*i*_ = 4π­(⟨*b*_*i*_^2^⟩ – ⟨*b*_*i*_⟩^2^) signifying the incoherent scattering cross section of the *i*-th atom, and *r*_i_(*t*) denoting the position of the *i*-th atom at time *t*. Coherent scattering probes the space and time correlations between atoms *i* and *j* over a time interval *t*, enabling the study of atomic structure and distinct motions. Conversely, incoherent scattering quantifies the time correlation of the *i*-th atom at a length scale defined by *q* over a time interval *t*, thereby providing insights into the self-motion of atoms.

A standard approach to measuring inelastic neutron scattering involves determining *E*_i_ and *E*_f_ over a specified range and calculating their difference. As the energy resolution of *E*_i_ (and/or *E*_f_) is enhanced, reduced values of *ℏω*, thus sensitive to QENS, are appreciated. Typically, a TOF-QENS machine covers energy ranges of sub meV to meV, and is sensitive to hydrogen motions in ps to 10s of ps.^71–73^ Conversely, NBS employs *E*_f_ ≈ 2 meV in conjunction with a range of *E*_i_ to probe *ℏω* with a high energy resolution of about 1 μeV (corresponding to the time resolution of about 1 ns).^74,75^ In NBS studies for PNCs, the primary focus is on the self-motion of H atoms in polymer chains. Consequently, the samples are meticulously prepared to ensure that incoherent scattering predominates within the designated *q* range.^68,70,76^

NSE is characterized by its ability to encompass the longest time scales of all neutron spectroscopies. It accesses the segmental to chain dynamics at small scattering angles in PNCs. The blending of polymers with varying deuteration levels results in a contrast in the scattering length density (SLD), $\mathrm{SLD}=\frac{\sum_{i}b_{i}}{V}$ , where *V* is the molecular volume, between hydrogenated and deuterated polymers, thereby generating coherent scattering at small scattering angles. In NSE, the Larmor precession of neutron spins in a magnetic field is employed to encode the energy change from the sample into precession angles.^77,78^ Consequently, the instrumental energy resolution becomes decoupled from the energy resolution of incoming neutrons. For a NSE experiment, a relatively large incoming energy distribution is employed. The final neutron spin state is indicative of the extent of energy transfer within the sample. Furthermore, as the spectrometer analyzes the spin state of the neutrons using a spin analyzer, the scattering intensity distribution is proportional to the dynamic structure factor weighted by the transmission of the spin analyzer, namely, $I_{\mathrm{NSE}}∼\int S\left(q,\omega \right)\frac{\left\langle 1+\cos\left(\omega t\right)\right\rangle }{2}d\omega =\frac{1}{2}\left[S\left(q\right)+S\left(q,t\right)\right]$ . The first term of the equation corresponds to the static structure factor while the second term is the intermediate scattering function. The dynamic range of a standard NSE spectrometer is 0.2 to 15 nm^–1^ in *q* and a few ps to 100s ns in time. NSE has been used to assess *S*_coh_(*q*,*t*) in PNCs,^68,70,76^ encompassing processes ranging from local segmental motions, Rouse to local reptation dynamics.^79,80^ The corresponding SANS intensity is proportional to *S*(*q*,*t* = 0).

### 2.2 MD Simulations

The computational approach employed to characterize hierarchical dynamics in PNCs relies on MD techniques spanning atomistic and coarse-grained resolutions. These simulations generate trajectories that sample equilibrium phase-space distributions, thereby providing direct access to dynamical correlation functions grounded in statistical mechanics. All-atom molecular dynamics (AMD) is a method that facilitates the analysis of polymer and NP interactions at the level of explicit atoms. This approach utilizes established force fields^34,81–83^ to define bonded and nonbonded interactions. The time evolution of the system is obtained by integrating Newton’s equations of motion,

5 $$m_{i}{\mathrm{r̈}}_{i}\left(t\right)=-\nabla_{i}U\left(r^{N}\left(t\right)\right)$$

using numerical integration algorithms such as velocity-Verlet under controlled thermodynamic ensembles.^84,85^ These atomistic simulations capture local packing, segmental orientation, and spatially heterogeneous mobility within interfacial regions, enabling the computation of short-time dynamical quantities. Among these, the self-part of the van Hove function,

6 $$G_{s}\left(r,t\right)=\left\langle \delta \left(r-\left|r_{i}\left(t\right)-r_{i}\left(0\right)\right|\right)\right\rangle$$

is employed to quantify the distribution of monomer displacements on ps to ns time scales. This method aligns with the statistical-mechanical definitions introduced by van Hove^86^ and discussed extensively in neutron scattering monographs.^65,87^ A recent review article by Arbe and co-workers highlights synergistic examples of integrated neutron scattering and MD for studying hierarchical structures and dynamics of polymer chains at different length and time scales (see Figure 3).^88^

**Figure 3:**
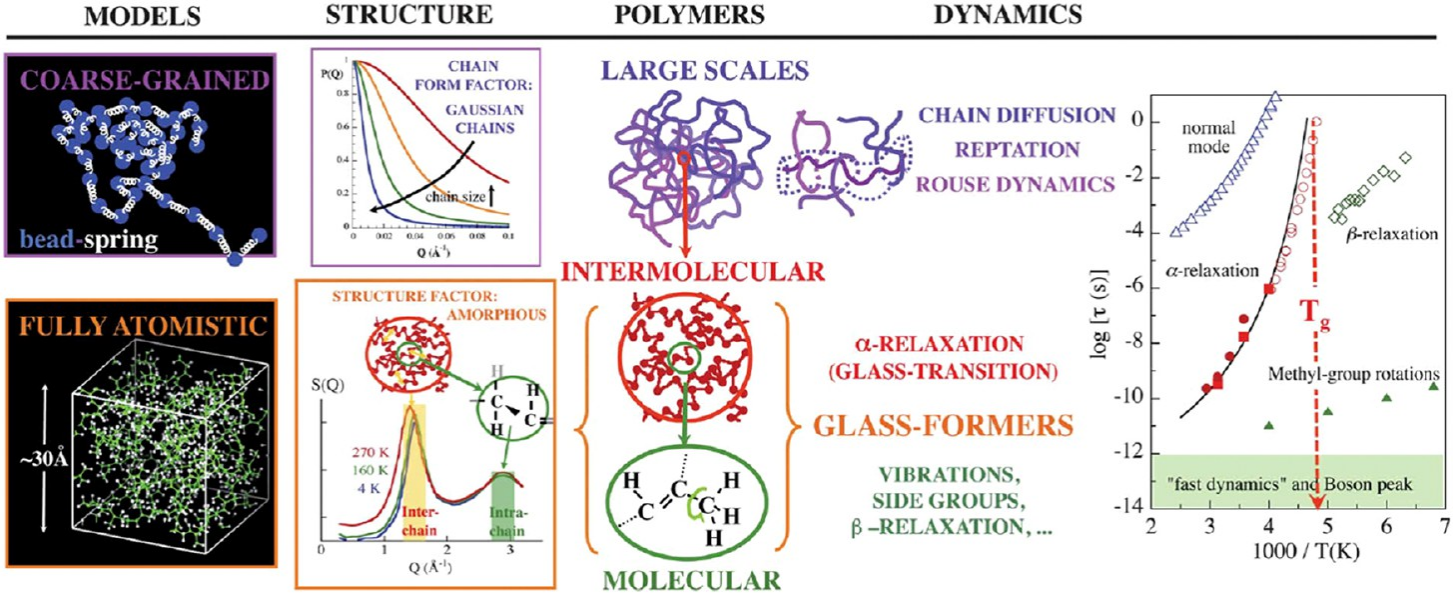
Integrated neutron scattering and MD methodologies to elucidate the relevant structures and dynamics of polymers as a function of the length scale of observation. Structure: chain form factor *P*(*q*) (schematic) and structure factor *S*(*q*) of polybutadiene dynamics: the different possible processes are listed and the relaxation map of polyisoprene obtained by dielectric spectroscopy (empty symbols) and neutron scattering (full symbols) illustrates some of them. At a given temperature, large-scale motions (e.g., the relaxation of the end-to-end vector of the chain monitored by the dielectric normal mode) display much slower characteristic times than the α-relaxation, which in turn is slower than side-group motions and vibrations. The left-hand images depict cartoons illustrating the appropriated types of simulations or models that are employed at different length scales. The figure is reproduced from ref 88 with permission from The Royal Society of Chemistry.

Coarse-grained molecular dynamics (CGMD) extends the accessible spatial and temporal scales by reducing the number of degrees of freedom through the mapping of multiple atoms to effective interaction sites. The resulting potentials, obtained through force-matching, relative-entropy optimization, or top-down parametrization, allow for the simulation of large polymer–NP systems over microsecond time scales.^85,89–96^ These models delineate chain-scale relaxation processes that govern mesoscale dynamics. Polymer motion in this regime is interpreted using classical models of polymer dynamics, including Rouse dynamics for unentangled chains and reptation theory for entangled systems.^79,80^ The coarse-grained representation also facilitates the calculation of the distinct part of the van Hove function,

7 $$G_{d}\left(r,t\right)=G\left(r,t\right)-G_{s}\left(r,t\right)$$

which captures the correlated displacements among particles and reflects cooperative behavior due to chain connectivity and NP-induced structural organization.

The full van Hove function, *G*(*r*,*t*) = *G*_*s*_(*r*,*t*) + *G*_*d*_(*r*,*t*), establishes a connection between MD simulations and neutron and X-ray scattering experiments. The spatial Fourier transform of the system under consideration yields the intermediate scattering function, *S*(*q*,*t*), defined as

8 $$S\left(q,t\right)=\int \mathrm{dr}e^{-\mathrm{iq}\cdot r}G\left(r,t\right)$$

representing the central dynamical observable in NSE spectroscopy.^87,97^ NSE spectroscopy quantifies the intermediate scattering function *S*(*q*,*t*), which is the time-domain Fourier transform of the van Hove correlation function. Conversely, NBS probes the frequency-domain dynamic structure factor *S*(*q*,ω). Note that the majority of NBS experiments capture the self-part of the van Hove correlation function through incoherent scattering as described in the previous section. The high-*q* behavior of *S*(*q*,*t*) is primarily reflective of local relaxation processes captured by AMD, whereas intermediate and low-*q* behavior is indicative of Rouse-mode decay and reptation-like motion accessible via CGMD.^88^

### 2.3 XPCS

XPCS,^98–101^ analog to dynamic light scattering (DLS) in the X-ray regime, measures fluctuations of the electron density distribution (i.e., X-ray contrast) of a sample in the time domain via time series of (partially) coherent scattering pattern *I*(*q*,*t*). Employing an X-ray beam of adequate transverse and temporal coherence^102^ results in a speckle pattern that reflects the coherent interference of all scatterers within the coherently illuminated scattering volume. Unlike in conventional scattering experiments, background subtraction for the speckle pattern is generally not feasible. Consequently, XPCS is sensitive to the electron density contrast that predominates the scattering signal. However, XPCS demonstrates compatibility with resonant or anomalous scattering, which can offer sensitivity to specific components within a sample. In polymeric systems, accessible time and length scales generally restrict experiments to the nano to mesoscale, corresponding to length scales of NPs and polymer networks.

These length scales are accessible via bulk-sensitive transmission (SAXS, see Figure 4) or surface/interface sensitive grazing incidence small angle scattering. The latter method may require a systematic consideration of reflection and refraction contributions to the XPCS signal.^103^ XPCS in grazing incidence geometry^99^ gives access to the dynamics in thin films, at surfaces^104^ and interfaces.^105^ Dynamic information is obtained from the scattering intensities *I*(*q*,*t*) via the normalized intensity–intensity correlation function

9 $$g^{\left(2\right)}\left(q,\tau \right)=\frac{{\left\langle I\left(q,t\right)I\left(q,t+\tau \right)\right\rangle }_{\mathrm{t̅},\mathrm{q̅}}}{{\left\langle I\left(q,t\right)\right\rangle }_{\mathrm{t̅},\mathrm{q̅}}^{2}}$$

where τ is the time bin (typically an integer multiple of the exposure time per frame in a continuous data acquisition) and the average ⟨···⟩_*t̅*,*q̅*_ over the duration of the measurement *t̅* and region of detector pixels with “equivalent” *q̅*. The advent of suitable pixelated detectors has led to the common characterization of dynamics via two-time (instantaneous) correlation functions, i.e.,

$$C\left(q,t_{1}{,t}_{2}\right)=\frac{{\left\langle I\left(q,t_{1}\right)\cdot I\left(q,t_{2}\right)\right\rangle }_{\mathrm{q̅}}}{{\left\langle I\left(q,t_{1}\right)\right\rangle }_{\mathrm{q̅}}\cdot {\left\langle I\left(q,t_{2}\right)\right\rangle }_{\mathrm{q̅}}}$$ 10

where the average ⟨···⟩_*q̅*_ does not include experimental time. Due to their time-resolved nature, two-time correlation functions are capable of describing out-of-equilibrium dynamics. The presence of dynamical heterogeneities can be detected by their normalized variance (χ),^106,107^ while the time scale of the temporal heterogeneity can be determined from the fourth order correlation function.^108^ One method of quantifying time scales from two-time correlation functions is via “aged” one-time correlation functions *g* ^(2)^(*q*, *t*_age_, τ) = ⟨*C*(*q*, *t*_1_, *t*_2_)⟩_*t*_age__, where $t_{\mathrm{age}}=\frac{\left(t_{1}+t_{2}\right)}{2}$ , and τ = |*t*_1_ – *t*_2_|. The average ⟨···⟩_*t*_age__ is taken over *t*_age_ with quasi-equilibrium dynamics.

**Figure 4:**
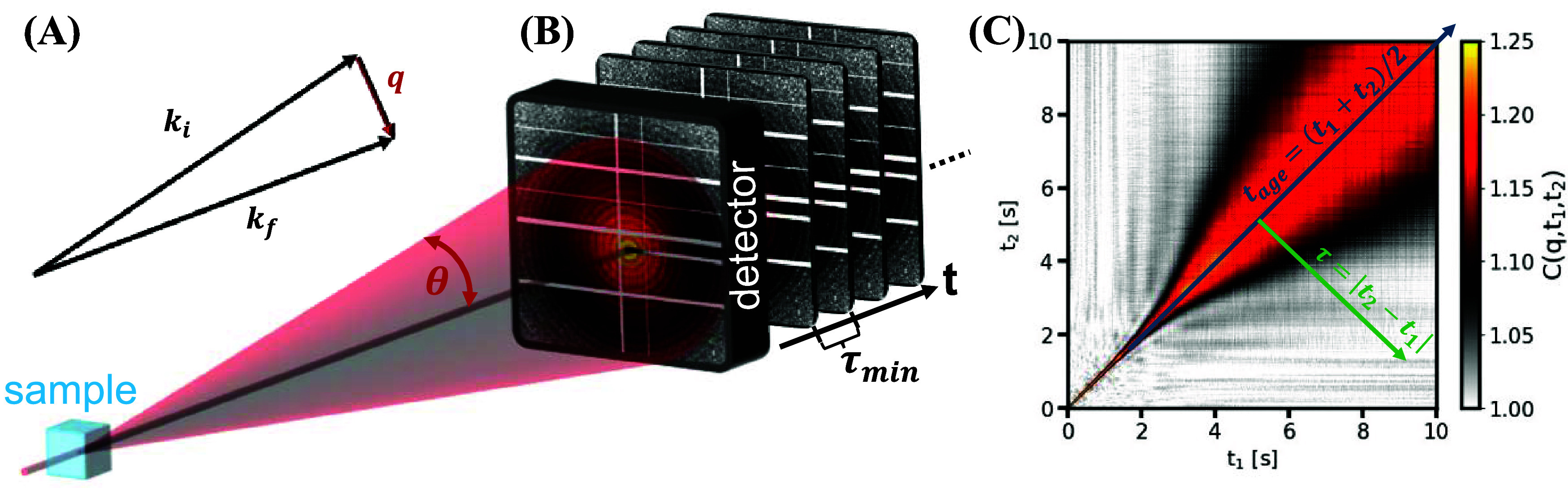
(A) wavevector transfer ***q*** = ***k***_*f*_ – ***k***_*i*_, with | ***k***_*i*_| = | ***k***_*f*_| for elastic scattering. For the transmission geometry depicted in (B), $\left|q\right|=\frac{4\pi }{\lambda }\sin\left(\theta /2\right)$ , with the X-ray wavelength (λ). The shortest lag time τ_min_ is the period between consecutive speckle pattern in the time series recorded by the detector. (C) example of a two-time correlation function showing out-of-equilibrium dynamics.

Under conditions of Gaussian statistics, the Sieger relation $g^{\left(2\right)}\left(q,\tau \right)=1+\beta_{0}\left(q\right){\left|\frac{S\left(q,\tau \right)}{S\left(q,0\right)}\right|}^{2}$ (with the setup dependent contrast factor β_0_(*q*) ≤ 1) connects *g* ^(2)^(*q*,τ) and the intermediate scattering function *S*(*q*,τ). The one-time correlation functions generally take the form *g* ^(2)^(*q*,τ) = 1 + β_0_(*q*)|*g* ^(1)^(*q*,τ)|, where β_0_(*q*) ≤ 1 is a setup dependent contrast factor and *g* ^(1)^ is the field correlation function proportional to the intermediate scattering function of the system. *g* ^(2)^ can often be described by the KWW form, which is a phenomenological model that is used to describe nonexponential relaxation processes in disordered systems:^107,109^

11 $$g^{\left(2\right)}\left(q,\tau \right)=1+\beta_{0}\left(q\right)\exp\left[-2{\left(\frac{\tau }{\tau_{0}\left(q\right)}\right)}^{\gamma \left(q\right)}\right]$$

with the *q*-dependent stretching exponent (γ < 1) or compression exponent (γ > 1), in addition to the characteristic time-scale τ_0_. The diffusion of spherical NPs can be categorized as either simple Brownian diffusion (γ = 1) or anomalous diffusion (γ ≠ 1). The former is characterized by τ_0_(*q*) = 1/(*D*_*f*_ *q* ^2^) with a diffusion constant *D*_*f*_, whereas the latter is described by the Continuous-Time Random walk model.^110^ In the case of more complex diffusive dynamics, typically involving spherical tracer NPs in a complex fluid, the mean squared displacement ⟨Δ*r* ^2^(τ)⟩ can be obtained as $g^{\left(2\right)}\left(q,\tau \right)=1+\beta_{0}\;\exp\left[-\frac{q^{2}\left\langle \Delta r^{2}\left(\tau \right)\right\rangle }{3}\right]$ . This is utilized as the fundamental principle for “micro-rheology”, a term which refers to the calculation of storage (*G*′) and loss modulus (*G*″) for a given system.^111,112^

As a scattering technique, XPCS provides information on length scales 2π/*q*, which may differ from macroscopic measurements or predictions by continuum theory. A notable example is the slowing down of NP diffusion on length scales comparable to the next nearest neighbor distance, a phenomenon known as “de Gennes narrowing”.^113,114^ Soft condensed matter systems, such as PNCs, often exhibit nondiffusive dynamics. For instance, the relaxation mechanism proposed by Cipelletti and co-workers,^115^ in which NPs and their neighboring NPs move collectively with a certain velocity. This leads to the proportionality relationship, i.e., τ_0_(*q*) ∼ *q* ^–1^ for all *q* greater than the inverse of the distance traveled by NPs under strain. Therefore, a proportionality constant in the linear relation of τ_0_(*q*) = (*v*_*d*_ *q*)^−1^ represent the ballistic motion of the scatterer as the “displacement velocity (*v*_d_)”.^115^ Such “collective” dynamics are frequently accompanied by compression exponents γ > 1 in eq 11, which are commonly attributed to a sudden release of topological constraints in the polymer matrix.^116^ In particular, *q*-independent compression exponents (γ = 1.5) have been associated with dipole stress relaxation.^117^ Such stress-relaxation processes have the potential to yield fully ergodic dynamics, i.e., *g* ^(2)^(*q*,τ)→1 for finite τ, even in fully developed solids.^115,118,119^ Contrary, γ < 1 has been linked to the existence of a distribution of relaxation processes with analogous time scales within the sample. It has been observed that certain PNCs manifest dynamics characterized by two distinctly disparate time scales. One such time scale, designated as the “fast mode”, is associated with the diffusion of NPs within the “cage” formed by their nearest neighbors. The other time scale, referred to as the “slow mode”, is associated with the diffusion of NPs out of the “cage”. In this case, eq 11 assumes a more intricate form, thereby delineating multimodal dynamics.^120,121^ The time scale of a fast, nonergodic dynamic mode may extend beyond experimental time frame. However, its presence can be discerned through the ergodicity plateau, defined as β­(*q*)/(β_0_(*q*)) < 1. This ratio quantifies the extent of structural arrest on fast time scales with β­(*q*)/(β_0_(*q*)) = *f*_0_^2^exp­(−(*q* ^2^ *r*_0_^2^/3)), where *f*_0_ is the fraction of NPs whose dynamics are localized in rapid motion, constrained to the localization length *r*_0_.^122,123^

## 3 Structures and Dynamics of BP in a Chemically Identical Polymer Matrix

### 3.1 Background about BP

The typical sizes of primary CB and SiO_2_ elastomers utilized in the manufacturing of automotive tires range from a few tens of nanometers to 100 nm. Therefore, in comparison to micrometer-sized particles, the surface area of NPs increases significantly, thereby emphasizing the significance of polymer-NP interfaces.^1,2,13^ In the case of SiO_2_-filled elastomers, since the chemical interaction between the hydrophilic SiO_2_ surface (polar with different silanol groups and adsorbed water) and elastomers is considerably weaker than that between CB and elastomers.^124^ Consequently, silane coupling agents (SCA), including sulfur-containing silane-based bifunctional coupling agents, are frequently compounded with SiO_2_-filled elastomers in the industry.^125^ This complicates the design of the polymer-NP interface.^1,2,13^ Baeza and co-workers utilized simplified industrial PNCs composed of SiO_2_ and uncross-linked styrene–butadiene-rubber chains bearing a singular graftable silanol end-function group.^126–128^ Their findings are intriguing to show that the rolling resistance were enhanced by the grafting of the polymer to the SiO_2_ surface in comparison to conventional SiO_2_-filled elastomers with SCA, thereby highlighting a vital role of polymer chains at the interface in controlling the tire performance.

As initially proposed by Stickney and Falb,^129^ the BP is a very thin polymer layer formed on a NP surface via van der Waals interactions and is resistant to dissolution even in a good solvent. The experimental difficulty of the BP at the buried interface, as well as the fact that the matrix is composed of the same component, have led to many discussions of polymer conformations of the BP from a simulation point of view.^32,36,130–137^ Notably, the findings of these studies have led to a critical conclusion that the BP consists of two different chain conformations: The “flattened chains” that constitute an inner higher density region of the BP and the “loosely adsorbed chains” that form an outer bulk-like density region.^130,131,135–137^ These observations are consistent with a previous nuclear magnetic resonance (NMR) result, as shown in Figure 5.^38^ A recent report by Saalwächter and co-workers^138^ has refined the glass transition temperature (*T*_g_)-gradient model in the BP layer by combining NMR and differential scanning calorimetry (DSC) with mixtures of SiO_2_ NPs and P2VP. Their experimental results have highlighted the necessity of integrating multiple probes (including computational simulations) for studying the BP layer. This motivated the neutron scattering and MD studies on the BP layer, as elaborated in Sections 3.4. and 3.5. Similar two-layer structures of BP were also identified in polymer thin films prepared on planar solid surfaces.^139–152^ Readers are encouraged to refer to review articles for the novel properties of BP on planar surfaces.^153,154^

**Figure 5:**
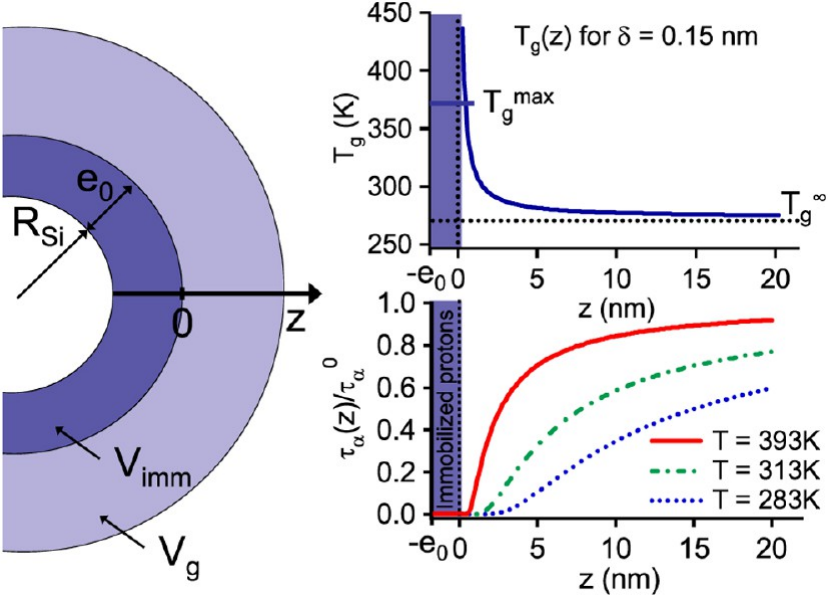
Top: Schematic representation of the glass-transition temperature gradient in the vicinity of a SiO_2_ NP, with a cutoff at *T*_g,max_. The volume *V*_min_ represents the immobilized protons at the surface of the NP, corresponding to a thickness *e*_0_. *V*_g_ the volume on which the *T*_g_ gradient is applied. Bottom: Variation of the ratio of the Williams–Landel–Ferry (WLF) time at a distance *z* over the WLF time in the bulk τ_α_(*z*)/τ_α_^0^ (the WLF coefficients take into account the *T*_g_ change at the NMR frequency). The figure is reproduced from ref 38 with permission from American Physical Society.

Twiss made the first observation in 1925 on the effect of BP of CB on improved mechanical properties of elastomers.^155^ Subsequent research has reached a consensus regarding the critical role of the BP in the reinforcement of CB-filled elastomers^24,26–28,30,48^ and SiO_2_-filled elastomers.^6,156^ According to the seminal work of Simha and co-workers,^157^ the equilibrium BP chain on a solid surface is composed of three distinct segment sequences (i.e., trains, loops, and tails). Trains are used to denote adsorbed segments. Loops refer to sequences of free segments that connect successive trains, tails are defined as nonadsorbed chain ends (see Figure 1C). In addition, Long and Lequeux proposed a thermodynamic model positing that the strong adsorption of polymer chains to a solid surface amplifies the percolation of small domains of slow dynamics relative to the bulk.^158^ These phenomena give rise to a complex hierarchy of structures and dynamics at the segment and chain levels, thereby introducing further complications in understanding the molecular mechanisms behind the reinforcement effect.

### 3.2 Experimental Strategies to Directly Probe BP

The utilization of NSE in conjunction with deuterium molecules, as well as the subsequent data analysis has been established by Richter and co-workers^15,76^ to either enhance or suppress the contributions of a relaxation signal from selected subunits of a given polymer system. It should be noted that only the motion of these structures that contribute to the SANS intensity is probed by the corresponding NSE experiments. In the context of single chain dynamics investigations of polymers in melts without NPs, a mixture of about 10% hydrogenated polymer (h-polymer) within a deuterated polymer (d-polymer) matrix is conventionally employed to optimize the intensity-to-background ratio, particularly in the range of *q* from 1 to 2 nm^–1^. Conversely, when NPs are present in a polymer matrix, a contrast matching experiment is preferred to avoid the contributions from NPs. In order to investigate the chain dynamics within the experimental NSE time window, it is essential to utilize relatively low-molecular-weight polymers (typically below 50 kg/mol). A methodology was developed to selectively probe BP chains separated from the matrix chains and further elucidate the effect of NP types on the structure and dynamics of BP chains in a chemically identical polymer matrix. To this end, monodisperse hydrogenated polybutadiene (hPB, weight-average molecular weight (*M*_w_) = 45.1 kg/mol, polydispersity index (*Đ*_*i*_) = 1.08, 1,4 addition = 80%, “hPB50k”) for BP and monodisperse deuterated polybutadiene (dPB, *M*_w_ = 50 kg/mol, *D*_*i*_ = 1.08, 1,4 addition = 80%, “dPB50k”) for a matrix were synthesized. It is important to note that the matrix is not cross-linked, given that the chain dynamics are frozen. The cross-link motions in cross-linked PB at the segmental level were recently reported by Richter and co-workers using wide angle spinecho (WASP), NBS, and neutron TOF spectroscopy.^159^

#### 3.2.1 CB NPs

For CB-filled nanocomposites, the neutron scattering length density (SLD_N_) of CB (SLD_N,CB_ ≈ 6.1 × 10^–4^ (nm^–2^)) is nearly identical to that of d-polymer (for instance, deuterated polybutadiene (dPB, SLD_N,dPB_ ≈ 6.5 × 10^–4^ nm^–2^)), allowing for the performance of contrast-matched experiments and the selective labeling of the BP layer, thereby creating strong contrast between the matrix and BP. The spherical CB NPs (Asahi Carbon Co., Japan, an average diameter of 86 ± 40 nm) were first dissolved in toluene and subsequently added to the hPB50k solutions, which are then sonicated for a duration of 1 h. Subsequently, the solvent was evaporated from the mixed solution, and the composite samples were subjected to annealing at 100 °C for 3 h. The CB-filled hPB50k compounds were then cut into minute pieces and ensconced within a metal wire mesh cage (illustration (i) of Figure 6). The cage was immersed in a sufficiently large amount of hydrogenated toluene (h-toluene) to remove soluble polymer components. The solvent leaching process was repeated at room temperature for several days until the weight of the CB with the insoluble polymer component (i.e., the BP layer) remained unchanged (illustration (ii) of Figure 6A). The resultant “BP coated-CB” were first immersed in h-toluene and dispersed using an ultrasonic bath for about 5 h (illustration (iii) of Figure 6A). Afterward, the solution was left on a table for a period to allow the incompletely coated CB to precipitate. The upper homogeneous portion of the solution containing the BP-coated CB was collected and subsequently separated by centrifugation (process “p” of Figure 6A). The series of operations was repeated multiple times to purify the final BP-coated CB. The resulting BP-coated CB solution was then mixed in a dPB50k/butylated hydroxytoluene (an antioxidant agent, with a mass fraction of 0.1%)/toluene solution. The solvent was then evaporated from the mixed solution, and the composite samples were further dried in a vacuum oven at 100 °C for an extended period of time (≈7 days) (illustration (iv) of Figure 6A). The BP-coated CB was mixed with dPB50k for SANS and NSE experiments (illustration (v) of Figure 6A). The BP-coated CB loading was adjusted to a volume fraction of 20%, which is below the percolation threshold (≈a volume fraction of about 30%),^18^ so that the effect of polymer bridges between adjacent NPs can be ruled out. The thickness of the BP (not including the matrix) was estimated to be about 5 nm based on the results of transmission electron microscopy (TEM) analysis (see Figure 6B).

**Figure 6:**
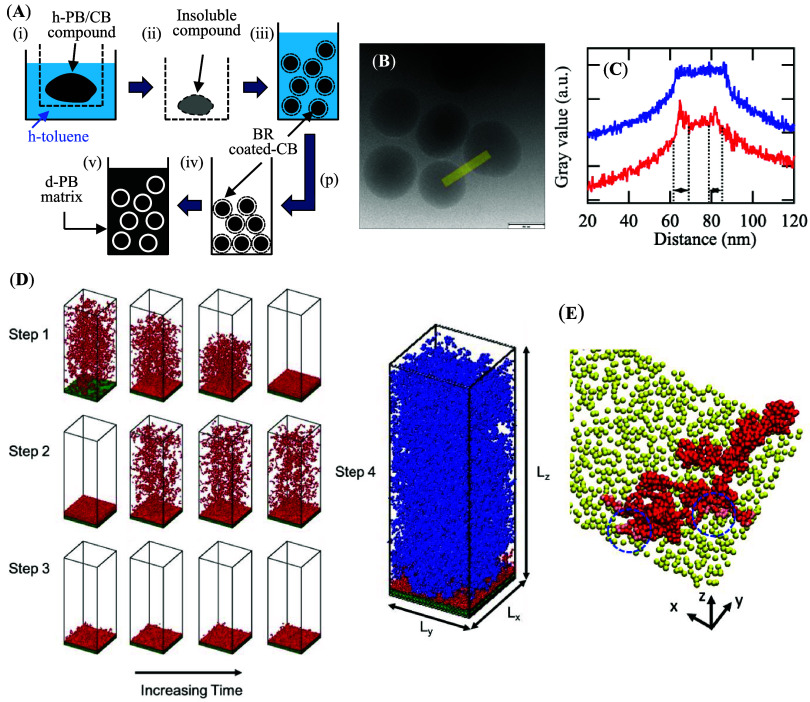
(A) Schematic diagram of the sample preparation protocol for the NSE and SANS experiments. (B) Zero-loss energy filtered TEM image of the BP-coated SiO_2_ in dPB50k after OsO_4_ staining. The scale bar corresponds to 90 nm. (C) Gray value profile along the yellow lines marked on the image (B) with an average width of 200 pixels show an increase in intensity in a region of 5 to 10 nm near the SiO_2_ surface for the OsO_4_-stained sample (represented in red). We assume that this is the BP (indicated by the regions between the two dotted lines), where the density is higher than the matrix or the packed chain conformation, makes it less accessible to staining. For comparison, the gray value profile for the BP-coated SiO_2_ in dPB50k prior to OsO_4_ staining is represented by the blue line. (D) Equilibration simulation steps for CB surface: (Step 1) Adsorption of polymers (red beads) onto a highly adsorbing substrate (green beads) and aided by compressing the top wall (not shown). (Step 2) Release of top-wall and desorption of weakly adsorbed chains. (Step 3) Removal of unadsorbed chains and equilibration of the bound polymer layer. (Step 4) Addition of polymer chains (blue beads) as a matrix in a simulation box with dimensions *L*_*x*_ = 40σ, *L*_*y*_ = 39.84σ, and *L*_*z*_ =(*m*_free_ *N*_free_)/(*cL*_*x*_ *L*_*y*_). (E) A single chain on the SiO_2_ surface is shown with pink beads (circled in blue dotted circles) representing monomers bonded to coupler beads used for CGMD simulations. Figures (A) and (D) are reproduced from ref 18, while figures (B), (C) and (E) are reproduced from ref 19.

#### 3.2.2 SiO_2_ NPs

In the context of SiO_2_-filled nanocomposites, the SLD value of SiO_2_ (SLD_N,SiO2_ ≈ 3.0 × 10^–4^ nm^–2^) exhibits an intermediate between those of hPB (SLD_N,hPB_ ≈ 0.4 × 10^–4^ nm^–2^) and dPB. Consequently, the design of contrast-matched neutron experiments necessitates the utilization of a blend comprising about 50% hPB and 50% dPB blend serving as the matrix. However, the mixture of hPB and dPB will yield a strong coherent scattering signal from the polymer chains. This is attributable to the significant SLD difference between them, which leads to substantial contributions from the dynamics of hPB in the matrix. Therefore, the employment of a hPB/dPB random copolymer with a deuteration level of about 50% is optimal.

In the case of SiO_2_ NPs, the chemical interaction between SiO_2_ (polar with different silanol groups and adsorbed water) and elastomers is much weaker than that between CB and elastomers.^124^ SCAs are typically compounded with SiO_2_-filled elastomers. Recent advancements in SCA designs for elastomers have focused on incorporating a triethoxysilyl moiety, which functions as a silica-binding group, and an appropriate organosulfur moiety. This organosulfur moiety can be attached to an unsaturated polymer chain via grafting to the sulfur atom.^125^ The experiments utilized a spherical SiO_2_ NP with an average diameter (*D*) of 112 ± 8 nm and the surface silanol density of 2.4 nm^–2^ (Nippon Shokubai). The error bars used in this paper represent ± 1 standard deviation unless otherwise noted. Bis­(3-triethoxysilylpropyl)­disulfide (SI266, (C_2_H_5_O)_3_Si­(CH_2_)_3_S_2_(CH_2_)_3_(C_2_H_5_O)_3_Si, Evonik) was used as the SCA. The quantity of SI266 was 8% by mass compared to the total mass of the SiO_2_ NP. This amount is optimized for industrial use.

The SiO_2_ NP and SI266 were initially dissolved in toluene and subsequently added to the hPB50k solutions, which were then sonicated for a duration of 1 h. The solvent was then evaporated from the mixed solution. To facilitate the silanization reaction on the NP surface^160^ and effectively work to strengthen the NP-polymer interface, the compounding condition at 140 °C for 1 h was used. In addition, this annealing condition converted at least 50% of the mercaptan to isolable disulfides and polysulfides,^161^ resulting in optimized sulfur vulcanization of elastomers for industrial use. The BP layer on the surface of the spherical SiO_2_ (hereafter referred to as “BP-coated SiO_2_”) was prepared by the aforementioned solvent leaching process. The NP loading was adjusted to a volume fraction of 20%, which is below the percolation threshold (≈30%).^19^ The thickness of the BP (without the matrix) was estimated to be 5.4 nm based on a thermogravimetric analysis experiment, while the thickness of the BP in the dPB50k matrix was determined to be about 7 nm based on TEM results (see Figure 6C).^19^ A PNC composed of SiO_2_, SI266, and dPB50k was utilized as a control (i.e., background). Subsequently, the scattering from the control sample was subtracted from that of the BP-coated SiO_2_ in dPB50k sample. This process allows for the elimination of scattering due to the contrast-mismatched conditions. The same background correction was applied to the BP-coated CB in dPB50k sample using a PNC composed of CB and dPB50k with 20% CB loadings.

It should be also emphasized that the SANS and NSE results for the contrast-matched system (i.e., a hPB/dPB random copolymer (*M*_n_ = 49.3 kg/mol, the composition of hPB/dPB = 53/47 (by mass)) as the matrix) were nearly identical to those for the noncontrast-matched sample (i.e., dPB50k as the matrix), with the exception of the scattering intensity. The noncontrast-matched sample exhibited a better signal-to-noise (S/N) ratio, attributable to the larger scattering contrast and low incoherent scattering signals.^19^ Therefore, it is reasonable to conclude that the cross-term contributions due to the contrast mismatch are minimal for the noncontrast-matched sample.^162^ Given that the complementary NBS technique primarily probes the hydrogen atom motions, the noncontrast-matched sample is more suitable for studying the structure and dynamics of the BP for the purpose of conducting a detailed SANS, NBS, and NSE data analysis.

### 3.3 Simulations Protocols

#### 3.3.1 CB NPs

The details of the CGMD simulation protocols for BP chains physically adsorbed on CB surfaces have been described elsewhere.^18,163^ Here we provide a brief overview of the strategic framework underlying the simulations. Given that the dimensions of CB NPs significantly exceed the BP thickness, the simulation box was modeled as a slab configuration,^163^ subjecting it to periodic boundary conditions in the *x* and *y* axes, while maintaining no restrictions along the *z* axis. An attractive substrate, composed of Lennard-Jones beads arranged in a hexagonal closed pack lattice, was positioned to act as a boundary at *z* = 0. The CB surface exhibits a high degree of roughness, as evidenced by its surface fractal topography.^164^ The surface features of the individual CB NP used for the neutron scattering experiments, with an average diameter of 86 nm, are expected to exhibit an order of magnitude smaller size (on the order of 1 nm) than the polymer itself.^165^ Consequently, the assumption was made that the adsorbing surface was smooth, thus neglecting the impact of surface roughness on polymer adsorption.

It is crucial to highlight the irreversible nature of polymer absorption onto the surface of NP, such as CB and elastomers, represented as 8 *k*_B_ *T*.^18,166^ To replicate the sample preparation that was employed for the neutron scattering experiments, the following simulation protocol was utilized to generate the bound chain on a CB surface. The procedure is composed of four fundamental steps (Figure 6D): (i) the preadsorption of polymer chains onto a highly adsorbing substrate, (ii) the subsequent removal of unadsorbed chains through a washing process, and (iii) the equilibration of bound polymer chains. The final step (iv) is to place the bound layer in contact with a polymer matrix. Fully flexible polymer chains with a degree-of-polymerization, *N*_*MD*_ = 2,000, which is in a similar order of magnitude compared to that of the experimental one (*N*_ex_ = 833), were utilized.

#### 3.3.2 SiO_2_ NPs

The simulation protocols used for the CB case were also applied to the SiO_2_ surface.^19^ However, we should consider the following key differences between the CB and SiO_2_. The surface of the SiO_2_ NPs is smooth and does not have a surface fractal structure. To mimic the actual SiO_2_ surface utilized for the neutron scattering experiments, grafting points of 0.6/σ^2^ (where σ is the diameter of a Lennard-Jones bead), which correspond to an actual silanol density of approximately 2.4/nm^2^ on the SiO_2_ surface used, were implemented. SCA molecules are represented as two connected beads (see Figure 6E), with one bead randomly grafted onto the surface at a grafting density of 0.3/σ^2^. This corresponds to a volume fraction of 50% at the NP surface, as determined by the independent X-ray reflectivity experiments using a BP layer prepared on a planar silicon with having a native oxide layer of 2.4 nm thickness.^19^ The system was constructed by introducing five hundred polymer chains with a degree-of-polymerization, *N*_MD_ = 1000. These chains were then placed on top of the substrate and silane couplers. The equilibration of the chain conformations was achieved using Monte Carlo bond swaps,^167^ which were implemented in LAMMPS.^168^ Subsequently, the monomers from the chains in close proximity to the silane couplers were then attached to the couplers until all free silane couplers were bonded to a chain.

At the chain level, the definition of a polymer bead as being adsorbed on to the NP surface is contingent upon its distance to a surface bead being less than 1.18 σ,^163^ which is slightly over 2^1/6^ σ. This is the minimum of the LJ 6–12 potential that describes the interaction between a polymer chain and substrate beads. A train is defined as a series of adsorbed connected beads. A tail is defined as a series of connected beads with one end adsorbed and the others unadsorbed. A loop is defined as a series of connected beads where the end beads are adsorbed beads. It is important to note that the repulsive nature of monomer-NP interaction in the PB/SiO_2_ system leads to the absence of a train, and the bound polymer layer on the SiO_2_ surface is characterized by the chains with at least one monomer bound to the NP surface.

### 3.4 Structures of BP

#### 3.4.1 SANS

The objective of the remaining portion of Section 3 is to ascertain the impact of NP types (with fixed NP loadings and analogous primary NP size and aggregation structures) on the structures and dynamics of the BP within the same polymer matrix and the corresponding property.

We first briefly mention the equilibrium structures of BP in the chemically identical polymer matrix using SANS, which is essential for the NSE/NBS data analysis. As shown in Figure 7A, the excess scattering from the BP on both the CB and SiO_2_ surfaces at 50 °C is shown after subtraction of the scattering contribution from the control samples. The excess scattering is attributable to the local density heterogeneity resulting from the penetration of matrix chains into the BP layer (i.e., polymer chain scattering). The figure indicates that the excess scattering profiles of the BP on the different NP surfaces exhibit a significant difference. To analyze the excess scattering, the following three assumptions were made:^18^ (i) the CB and SiO_2_ NPs are approximated as a planar geometry (see Section 3.3); (ii) the BP is modeled as two layers, an inner layer composed of strongly adsorbed chains and an outer layer composed of loosely adsorbed chains (see Section 3.1); (iii) the concentrations of loosely adsorbed chains composed of tails and loops of a BP chain can be estimated using a parabolic function reported for a polymer brush in a polymer melt.^169^

**Figure 7:**
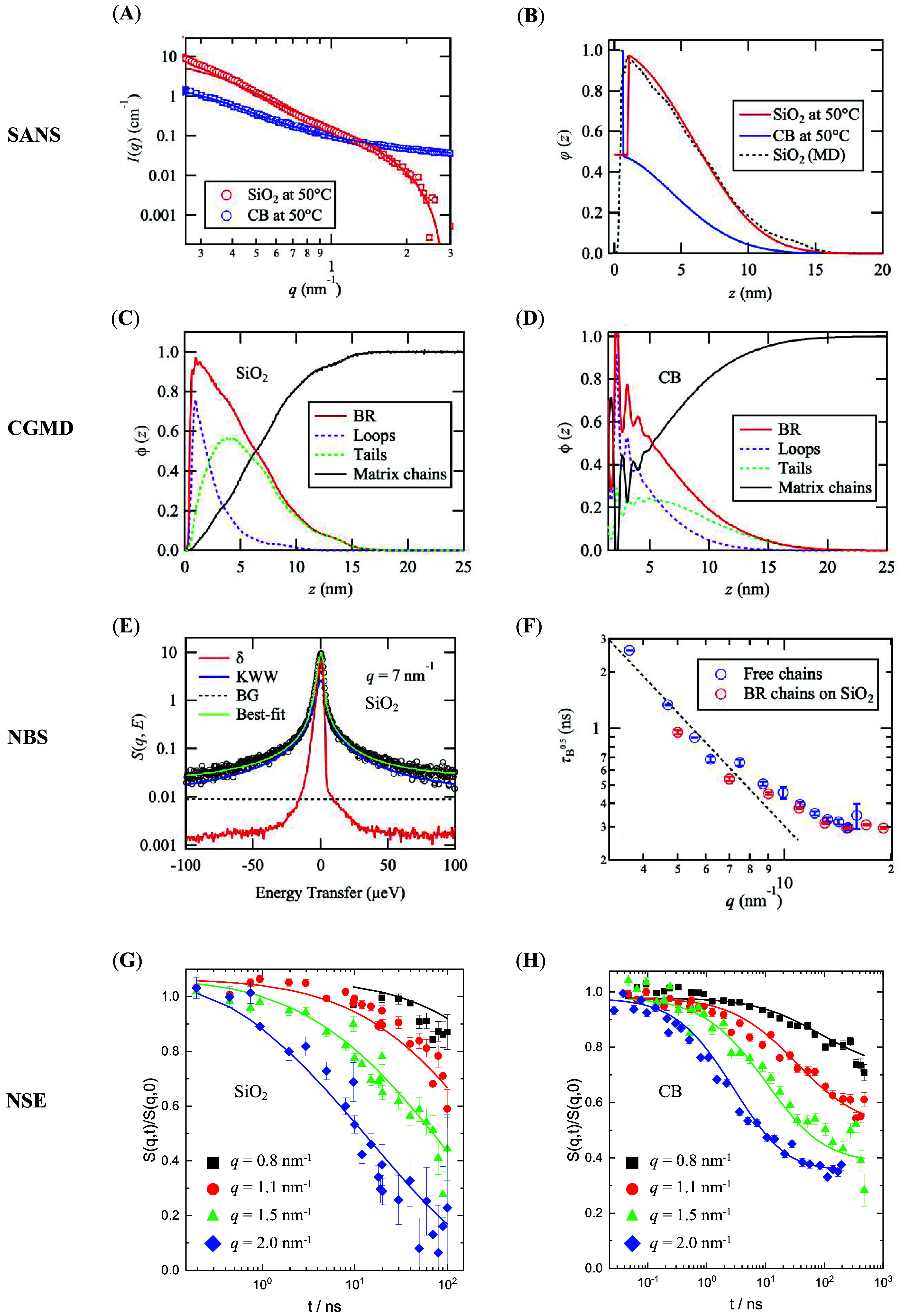
(A) Excess scattering from the BP on the SiO_2_ and CB surfaces at 50 °C after the background corrections. The best-fit of the density model described in the main text to the data is represented by the solid line. (B) Polymer volume fraction profiles of the BP layer on both the SiO_2_ and CB surfaces as a function of the distance from the NP surface (*z*) obtained from the SANS results. The dotted line corresponds to the MD results with the length conversion of 1σ = 0.41 nm. Volume fraction profiles of loops, tails, total (loops and tails, labeled as “BR”), and free matrix chains along the *z*-direction: (C) SiO_2_ and (D) CB. For the CB systems the train distributions are not shown for simplicity. The “BR” in the figure is the sum of loops, tails, and trains. (E) NBS spectra (circles) for the BP-coated SiO_2_ in dPB50k at *q* = 7 nm^–1^ and 27 °C. The solid line (shown in green) corresponds to the best-fit of eq 12 to the NBS spectra. (F) Segmental relaxation times (τ_B_) for the free and bound chains (identified as “BR”) with the value of the stretch exponent γ = 0.5 for the KWW function at 27 °C. For *q* < 8 nm^–1^, the power-law behavior τ_B_^0.5^ ∼ *q* ^–2^ (indicated in the dotted line) holds. (G) *S*(*q*,*t*)/*S*(*q*,0) for the BP-coated SiO_2_ in dPB50k at the given four *q* values (*q* = 0.8 nm^–1^ (black), *q* = 1.1 nm^–1^ (red), *q* = 1.5 nm^–1^(green), and *q* = 2.0 nm^–1^(blue)) and 150 °C. The solid lines correspond to the best fits of eq 13 to the data. (H) *S*(*q*,*t*)/*S*(*q*,0) for the BP-coated CB in dPB50k at the same four *q* values (*q* = 0.8 nm^–1^ (black), *q* = 1.1 nm^–1^ (red), *q* = 1.5 nm^–1^ (green), and *q* = 2.0 nm^–1^ (blue)) and 150 °C. The solid lines correspond to the best fits of eq 15 to the data. The Rouse rates (*Wl* ^4^) for the BP on the SiO_2_ and CB surfaces at 150 °C are fixed to the bulk (*Wl* ^4^ = 2.25 (nm^4^/ns)^18^), which was elucidated by the NBS results. Figures (A)–(H) are reproduced from ref 19.

Figure 7B shows the polymer volume fraction profile of the BP on the CB and SiO_2_ surfaces. The thickness of the chemisorbed BP (*L̅* = 10.0 ± 0.6 nm) on the SiO_2_ surface is nearly equivalent to that of the physisorbed BR chains on the CB surface (Figure 7B). Therefore, the important conclusion is that the BP chain, whose thickness is approximately equivalent to 1.25 *R*_g_ (*R*_g_ of hPB50k is 7.9 nm^18^), is not fully stretched at the NP interface,^63^ irrespective of chain adsorption, whether chemical or physical. On the other hand, the overall volume fraction profile of the BP on the SiO_2_ surface exhibits notable difference compared to that on the CB surface. The result indicates the absence of an inner layer composed of trains adjacent to the SiO_2_ surface, which does not allow the penetration of matrix chains into it. This phenomenon is identified in the physisorbed BP chains on the CB surface (Figure 7B).^18^ Instead, the presence of a SCA monolayer (≈1 nm thick) is identified at the SiO_2_ surface.^19^ In addition, the interfacial roughness (σ_s_ = 3.0 ± 0.5 nm) at the BR and matrix at the SiO_2_ surface is considerably smaller than that at the CB surface (σ_s_ = 4.9 ± 0.5 nm),^18^ suggesting a weaker adhesion between the BP and the free chains for the SiO_2_-filled elastomer. Furthermore, it is crucial to underscore that the structural integrity of the BP layer on both NP surfaces remained unaltered across a temperature range extending from room temperature to 150 °C. This temperature range far exceeds the bulk *T*_g_ (≈−90 °C).^18,19^

#### 3.4.2 CGMD

To provide further insight into the structural information about the BP layer on the CB and SiO_2_ surfaces, CGMD simulations for polymers adsorbed onto an impenetrable attractive substrate (representing CB) and an impenetrable repulsive substrate (representing SiO_2_) in a polymer melt are discussed. As shown in Figure 7C and D, the volume fraction profiles of the BP chains are depicted as a function of *z* from the SiO_2_ surface^19^ and from the CB surface.^18^ The CGMD results, with the length conversions of 1σ ≈ 0.41 nm for the SiO_2_ and 1σ ≈ 0.7 nm for the CB, are in good agreement with the SANS results. The loops on the SiO_2_ surface are located in proximity to the SCA/SiO_2_ surface, while the tails are presumably expelled from the SiO_2_ surface. Conversely, the loops on the CB surface extend from the CB surface, thereby enabling the penetration of the free chains into them, even in close proximity to the CB surface. This is a sharp contrast in terms of the BP chains between the SiO_2_ and CB surfaces, and it is key to the formation of an adhesive interphase between the NP and matrix polymer. The volume fraction profile of the matrix chain for the SiO_2_ NP is also plotted in Figure 6C. The presence of small loops, with an average height of approximately 2 nm, has been demonstrated to impede the penetration of matrix chains.^19^

### 3.5 Hierarchical Dynamics of BP

#### 3.5.1 Segmental Dynamics of BP

As demonstrated in Figure 7E, the representative dynamic structure factor, *S*(*q*, *E*), which was obtained by NBS experiments, for the BP-coated SiO_2_ in dPB50k at *q* = 7.0 nm^–1^ and *T* = 27 °C is presented. The data were corrected for background subtraction using the SiO_2_/SI266 in dPB50k sample. Richter and co-workers reported that the intermediate incoherent scattering function attributed to the segmental relaxation in polymers above the *T*_g_ is well described by the Kohlrausch–Williams–Watts (KWW) equation.^170^ However, it was determined that the contribution alone was not sufficient for the adequate expression of the experimental data. Consequently, a minor elastic peak contribution was taken into consideration. This phenomenon may be attributed to the presence of a silica background.^171^ Therefore, we approximate *S*(*q*, *E*) for the BP-coated SiO_2_ in dPB50k as the sum of the KWW equation and the elastic contribution, *i.e*.,

12 $$S\left(q,E\right)=(A_{KWW}F\left[\exp\left[-{\left(\frac{t}{\tau_{B}\left(q\right)}\right)}^{\gamma }\right]\right]+A_{d}\delta \left(E\right))⊗R\left(q,E\right)+\mathrm{BG}$$

where τ_B_(*q*) is the segmental relaxation time, γ is the stretching exponent, *A*_KWW_ and *A*_d_ (*A*_KWW_+*A*_d_ = 1) are numerical constants, δ­(*E*) is a delta function that is approximated to express an elastic contribution, and BG is the constant background. The symbol F denotes the Fourier transform, *R*(*q*,*E*) is the resolution function, and ⊗ represents a convolution operator. The exponent of γ = 0.5 for the segmental dynamics in polymer melts is predicted by the standard Rouse model.^172^ As shown in Figure 7E, eq 12 exhibited a high degree of agreement with the *S*(*q*, *E*) for the BP-coated SiO_2_ in dPB50k with γ = 0.5.

Figure 7F shows the *q*-dependence of τ_B_(*q*) for the BP-coated SiO_2_ in dPB50k. To facilitate a meaningful comparison, the *q* dependence of τ_B_(*q*) for the free hPB50k chains in the dPB50k matrix was measured.^18^ The volume fraction of hPB50k was set to 3.1% by volume, which is equivalent to that used for the BP-coated SiO_2_ in dPB50k. The findings of the study demonstrated that the segmental motions between the chemically grafted bound chains and the free chains in the melts remain constant. Concurrently, the segmental dynamics of BP on the CB surface exhibited identical characteristics to those of the free chains in the melts. Consequently, it was determined that the average segmental dynamics of the BR chains on the SiO_2_ and CB surfaces, where a comparable degree of chain stretching is evident (Figure 7C and D), are not influenced by chain adsorption either chemically or physically. This outcome aligns well with experimental findings on other polymer nanocomposite systems,^53,171^ while simulation studies^173,174^ indicated a gradient of segmental motions when the polymer-NP interaction is attractive (e.g., a PB/CB blend system).

To further elucidate the dynamics of the interface, the mean square displacement (*MSD*) of BP chains was examined, with the data derived from CGMD simulations on the CB surface. For a more thorough discussion of this topic, please refer to ref 18. The presence of immobile trains on the CB surface was evident, thereby giving rise to the elastic contribution identified at *q* > 10 nm^–1^ within the NBS time domain. It was also concluded that the MSD of the mobile tail and loop components at 0.7 σ < *z* < 1.7σ (1σ ≈ 0.7 nm) within the relevant NBS time window is approximately three times smaller than the bulk, while that at 1.7 σ < *z* < 2.7σ almost recovers the bulk behavior. Consequently, a local heterogeneity in the segmental dynamics within such a confined space (∼2 nm from the CB surface) occurs due to chain binding. As illustrated in Figure 7D, the proportion of loops and tails within a thickness of about 2 nm is estimated to be 16% of the total loop and tail components. Therefore, it can be deduced that the quasielastic scattering we measured by NBS is representative of the sum of the *MSDs* of the tail and loop components at *z* < 2 nm and the MSDs at *z* > 2 nm (which can be approximated as the *MSD* of the bulk) by their compositions. This observation is consistent with the NBS results, thereby validating the reliability of both the measurement and computational methods.

For SiO_2_-filled PNCs, BDS has been extensively utilized to examine the segmental dynamics of polymer chains (see review articles^17,20^ ). BDS measures fluctuations of dipole moments and diffusion of charges, covering an extremely broad frequency range (12 to 15 decades) from several μHz to above several GHz. The corresponding time scale ranges from a few ps to hours. Genix and co-workers^17,20^ have demonstrated that BDS is a powerful approach to discuss the segmental dynamics of the BP as well as its thickness and the volume fraction with respect to the total polymer volume.^175^ Notwithstanding the inherent spatial resolution limitations, which yield merely average information across the entire BP layer, the integration of other laboratory-based techniques, such as SAXS, sum frequency generation, and X-ray photoelectron spectroscopy,^176^ can provide novel insight into the chain packing at the polymer-NP interface and motivate scientists seeking more accessible alternatives for PNC study.

#### 3.5.2 Chain Dynamics of BP

Subsequently, the focus will shift to the dynamics of the BP at the chain level. Numerical studies have demonstrated that NSE is an optimal instrument for examining the chain dynamics of unentangled chains in the melt (i.e., the Rouse dynamics^76^). The Rouse dynamics model the dynamics of a Gaussian chain in a heat bath driven by the entropic force resulting from the conformational chain entropy. The model is solved by modes *p* along a chain with mode relaxation times (τ_p_) and corresponding wavelength of *Nl*/*p* (*N* and *l* are the number of segments in a chain and the segment length, respectively). Here the term τ_p_ is defined as τ_p_ = *R*_*e*_^4^/(*Wl* ^4^π^2^ *p* ^2^), where *R*_e_ is the chain end-to-end distance and *Wl* ^4^ denotes the elementary scale-independent Rouse rate, which characterizes the relaxation rate of a segment length. The latter is defined as *Wl* ^4^ = 3*k*_B_ *Tl* ^2^/ζ (*k*_B_ is the Boltzmann constant, *T* is the absolute temperature, and ζ is the monomer friction coefficient). Therefore, the normalized intermediate dynamic structure factor is defined as

13 $$S\left(q,t\right)/S\left(q,0\right)=\sum_{m,n}^{N}\exp\left(-\frac{1}{6}q^{2}B\left(n,m,t\right)\right)$$

where *B*(*n*,*m*,*t*) is the mean square segment correlation function. The function *B*(*n*,*m*,*t*) is defined as follows:

$$B\left(n,m,t\right)={6D}_{R}t+\left(m-n\right)l^{2}+\frac{{4R}_{e}^{2}}{\pi^{2}}\overset{N‐s}{\underset{p=1‐s}{\sum }}\frac{1}{p^{2}}\left\{\cos\left(\frac{p\pi m}{N}\right)\times \cos\left(\frac{p\pi n}{N}\right)[1-\exp\left(-\frac{t}{\tau_{p}}\right)]\right\}$$ 14

where *m* and *n* represent the segment indices, and *D*_R_ = *Wl* ^4^/(3*R*_*e*_^2^) is the overall diffusion coefficient. In the case of a chain in the melt, *s* = 0. Conversely, for an end-fixed chain, whether through physical or chemical means, *s* = 1/2 and *D*_R_ = 0.^171^

It has been demonstrated that anchoring points can suppress the mode amplitudes of the long-wavelength Rouse modes, a phenomenon referred to as “chain confinement”.^177^ In other words, the effective confinement is achieved through the suppression of the center-of-mass diffusion and the elimination of modes with wavelengths longer than the distance between anchoring points, as outlined in eq 14.^178^ In the case of a pseudobrush, Krutyeva and co-workers also considered anchored chains with a different number of segments (*N*_sub_) between anchor points and/or tails. This objective was accomplished by implementing a smoothly varying cutoff function (modeled by a Fermi function) with an additional parameter, designated as Δ*p*, which represented the width of the transition region. The detailed data analysis employing the modified Rouse model has been described elsewhere.^171,177^ It should be noted that, in the case of the BP on the CB surface, there are the elastic contributions from CB, as well as the immobile trains in the *q* range employed for NSE, as previously reported in the BP chains on the SiO_2_ surfaces in unentangled poly­(ethylene glycol) and poly­(butylene oxide) matrices.^179^ Accordingly, we have modified eq 13 as follows:

15 $$S\left(q,t\right)/S\left(q,0\right)=\mu +\left(1-\mu \right)\times \sum_{m,n}^{N}\exp\left(-\frac{1}{6}q^{2}B\left(n,m,t\right)\right)$$

where μ is the fraction of immobile polymer components.

As illustrated in Figures 7G and H, the *S*(*q,t*)/*S*(*q*,0) for the BR-coated SiO_2_ in dPB50k and for the BR-coated CB in dPB50k are exhibited at the four different *q* values and 150 °C. The modified Rouse model was applied to the NSE data for the BP chains on the CB and SiO_2_ surfaces. The solid lines in Figure 7G and H represent the best fits of the suppressed Rouse model to the NSE data. The findings indicate that the overall behavior of *S*(*q,t*)/*S*(*q*,0) can be adequately elucidated by the existence of mode restrictions with multiple anchoring points (*p* ≈ 52 and Δ*p* ≈ 30) for the BP-coated SiO_2_ in dPB50k and *p* ≈ 8, Δ*p* ≈ 2, and μ = 0.35 for the BP-coated CB in dPB50k. The significant value of *p* for the BP chains on the SiO_2_ surface suggest a notable size distribution of loops, also known as confinement sizes. When *p* ≈ 52, the wavelength corresponds to *N*_sub_ ≈ 15 monomers. For the BP chains on the CB surface, *p* ≈ 8 leads to *N*_sub_ = 93. The end-to-end distance of a subchain corresponding to *N*_sub_ yields an effective loop size, $R_{\mathrm{ee}}=\sqrt{N_{\mathrm{sub}}l^{2}}$ (*l* = 0.67 nm for PB^18^). Consequently, the mean loop size on the CB surface is considerably larger (about a factor of $\sqrt{93/15}\approx 2.5$ ) than that on the SiO_2_ surface.

In summary, as illustrated in Figure 8A and B, the integration of neutron scattering methods and CGMD results leads to the following conclusions: The loop size of the BP on the SiO_2_ surface is, on average, considerably smaller than that on the CB surface. Additionally, the loops of the BP on the SiO_2_ surface exhibit significant crowding. Consequently, these characteristics of the loops impede the interdigitation of the BP on the SiO_2_ surface with the free polymer chains in the matrix.

**Figure 8:**
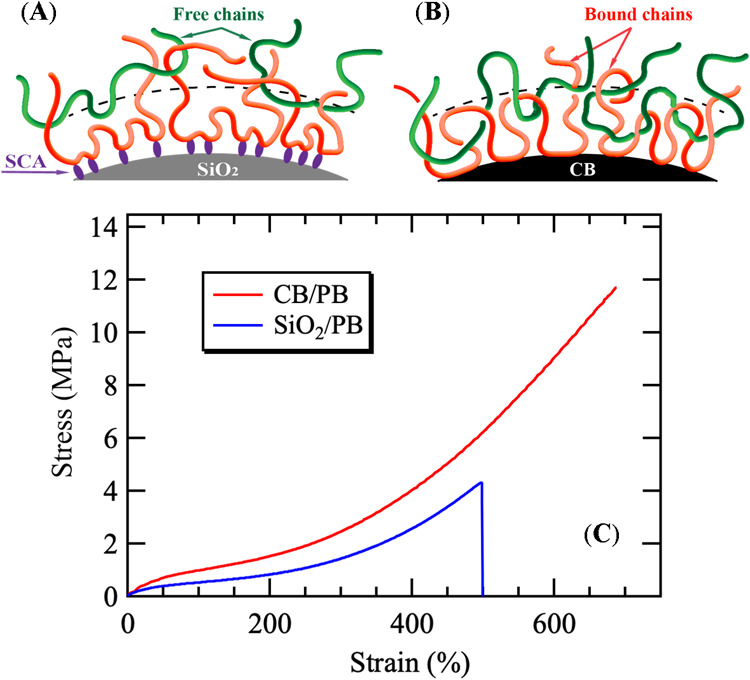
Schematic chain conformations of the BP on the (A) SiO_2_ surface and (B) CB surface. The BP chains are denoted by the color red. The purple elongated circles correspond to the SCA molecules, and the free chains in a polymer matrix are shown in green. For simplicity, trains (an immobile fraction of the BP chains) on the CB surface are omitted. (C) Stress–strain curves for the SiO_2_/hPB and CB/hPB blends. It should be noted that the BR-coated SiO_2_ in dPB50k and the BR-coated CB in dPB50k, utilized for the neutron experiments, were viscous liquids, rendering them impractical for stress–strain measurements. Given the widely acknowledged fact that the reinforcement effect of SiO_2_-reinforced elastomers is significantly weaker than that of CB-reinforced elastomers, and, that for car tires, CB or SiO_2_ are compounded into high molecular weight elastomers, the stress–strain curves were measured for the SiO_2_ and CB used for the main text, but different hPB (*M*_w_ = 500 kg/mol, *M*_w_/*M*_n_ = 3.1, Ube Industries, Japan). The volume fractions of the SiO_2_ and CB were also set at 20%. In addition, to mimic the real system, sulfur (a mass fraction of 1.6%) and vulcanizing agents (stearic acid, N-t-butyl-2-benzothiazole sulfonamide (a mass fraction of 1.0%), and biphenyl guanidine (a mass fraction of 1.0%)) were added to the compounds, resulting in the same degree of polymer network by cross-linking (the mass ratios in parentheses are relative to the mass of hPB). Figures (A)–(C) are reproduced from ref 19.

### 3.6 Correlation between Interfacial Structure/Dynamics and Material Performance

As shown in Figure 8C, a CB/PB elastomer typically exhibits greater toughness and flexibility in comparison to a SiO_2_/PB elastomer, even in the absence of a NP network. The following question is posited: What are the respective roles of the three segmental components of the BP in the macroscopic property? As mentioned above, the presence of immobile trains on the CB surface has been observed,^18^ which may correspond to the so-called “glassy layer” reported in other PNCs.^180,181^ These trains have been shown to function as robust anchoring points for BP during deformation.^182^ However, given that the immobile inner layer constitutes a mere fraction, about 1%, of the CB diameter, this increase in the effective diameter of the CB plays a nonessential role in determining the storage modulus. Furthermore, the contribution of the immobile inner layer to the stress increase is predicted to remain constant irrespective of the strain amplitude.^183^ Consequently, the immobile inner layer appears to be unrelated to the substantial stress increase that has been observed at large strains. Further research is necessary to establish a strong correlation between the immobile layer identified by neutron scattering and the predicted glassy layer.

Subsequently, the interdigitation of tails with the matrix polymer chains is discussed. This is a critical component for the robust mechanical performance of PNCs, irrespective of polymer-NP interactions.^184,185^ The results of the CGMD simulations indicated that the mole fraction of tails (≈70%) in the bound chain on the SiO_2_ surface is dominant over the loop molar fraction (≈30%).^19^ Furthermore, the effective grafting density of the tails on the SiO_2_ surface (0.15 nm^–2^)^19^ is significantly higher than that on the CB surface (0.008 nm^–2^).^18^ However, as demonstrated in Figure 8C, the contribution of the tails to the mechanical property enhancement is negligible.

Finally, the role of loops in the interdigitation with matrix chains, another key to improving the mechanical modulus of PNCs, especially during stretching,^186^ is discussed. The CGMD results demonstrated that the grafting density of the loops on the SiO_2_ surface is several times higher (3.55 nm^–2^) than that of the loops on the CB surface (0.55 nm^–2^).^19^ This finding indicates an increase in the density and bulk/shear modulus of the BP on the SiO_2_ surface.^187^ However, these contributions are not associated with the material stress of the SiO_2_-filled elastomer depicted in Figure 8C. The integrated neutron scattering and CGMD results demonstrate that the substantial, uncrowded loops formed on the CB surface enable the interdigitation with the matrix polymer chains, even in proximity to the CB surface. This process results in the formation of an adhesive interphase between the BP and the polymer matrix, thereby enhancing the material’s performance. This concept is analogous to the improvement of the mechanical properties of PNCs through the incorporation of short chains, which function as a good solvent for long chains. The incorporation of short chains has been demonstrated to promote the formation of large loops by decreasing the proportion of trains.^188^

In the tire industry, hydrated amorphous SiO_2_ NPs are conventionally utilized. The NP–NP interaction in a SiO_2_ compound exhibits greater potency in comparison to CB-filled elastomers, attributable to the presence of polar groups in the unmodified, commercially available industrial grades of the precipitated silica surface. Consequently, the utilization of SCA is instrumental in the surface medication of SiO_2_. In addition to SI266, bis­(3-triethoxysilylpropyl)­disulfide ((C_2_H_5_O)_3_Si­(CH_2_)_3_S_4_(CH_2_)_3_(C_2_H_5_O)_3_Si, commonly referred to as SI69) has been utilized as an effective SCA to improve the reinforcement effect in the rubber industry.^189^ Recently, 3-octanoylthio-1-propyltriethoxysilane ((C_2_H_5_O)_3_Si­(CH_2_)_3_S­(CO)­(CH_2_)_6_C_2_H_3_), commonly known as NXT) and mercaptopropyl trimethoxysilane (HS­(CH_2_)_3_Si­(OCH_3_)_3_, commonly known as MPTMS) have become commercially available. It has been demonstrated that these SCAs possess the capacity to significantly enhance the performance of SiO_2_-filled elastomers, including dynamic properties, rolling resistance properties, and wet grips, when compared to SI266.^189,190^ However, the underlying mechanism responsible for this enhancement remains to be elucidated. It is hypothesized that the interfacial structure and dynamics of BP via NXT molecules promote the formation of large loops on the SiO_2_ surface, as revealed on the CB surface. Further research is necessary to develop for the development of the molecular design rules at the interface and achieve stronger material performance for SiO_2_-filled elastomers. The integration of a suite of neutron scattering techniques in conjunction with isotope labeling and MD calculations will undoubtedly facilitate the achievement of the aforementioned objective.

## 4 Structures and Dynamics of NP networks

### 4.1 Background

In the context of automobile tires, high NP loadings—typically about a volume fraction of 20%, as compared to a matrix polymer—are generally required. At such high NP loadings, chain confinement induced by neighboring NPs emerges,^51^ and the interparticle distance (ID)^191,192^ between neighboring NPs becomes a critical parameter in understanding the reinforcement. Colby and co-workers^61^ posit that when ID/2*R*_g_ = 1 (where *R*_g_ is the polymer radius of gyration), a flexible “rubbery bridge” is formed (see Figure 9A). This results in a NP network that leads to a most significant improvement in the mechanical properties.^57,61,193^ Recent reports^59,194^ have shown that the contributions of a percolated NP network to reinforcement extend beyond hydrodynamic (volumetric) arguments.^195–197^

**Figure 9:**
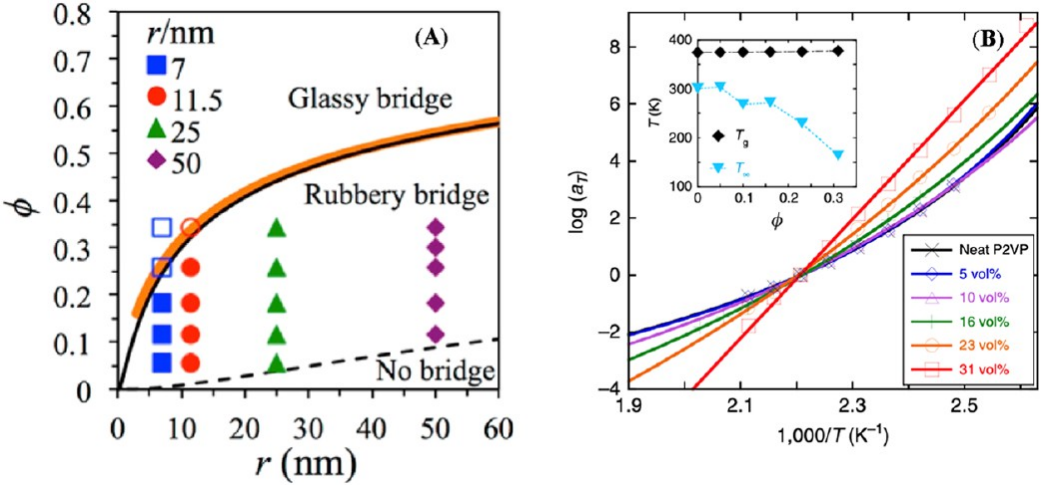
(A) NP concentration (ϕ) plotted against particle sizes *r* (symbols) for SiO_2_/P2VP (*M*_w_ = 97 kg/mol) nanocomposites at 180 °C. The phase diagram is drawn by the linear viscoelastic response of the nanocomposites. The phase diagram shows that small NPs are needed to achieve the stronger reinforcement from glassy bridges at reasonable particle loading. The figure is reproduced from ref 61. (B) Temperature dependence of the frequency-scale shift factor (*a*_T_) used to build the viscoelastic master curves for the neat P2VP (*M*_w_ = 554 kg/mol) matrix and its associated nanocomposites. Solid lines are WLF fits to the data, except for the 31 vol % data, which is fit to an Arrhenius equation. Inset: NPs volume fraction dependence of the glass transition temperature (*T*_g_) and Vogel temperature (*T*_N_) for the same samples. *T*_N_ has been extracted from fitting the data of the main figure with the Vogel–Fulcher–Tammann equation. The figure is reproduced from ref 199.

At further higher NP loadings, where NPs become crowded and the ID is comparable to the Kuhn length of polymer chains, a stiff “glassy bridge” forms (see Figure 9A).^61,198,199^ The glassy bridge is not intended to be permanent; it is susceptible to fracture when subjected to applied strain or at high temperatures.^57^ This susceptibility is likely to influence the Payne effect^200^ or the temperature dependence of a reinforcement effect.^59^ Baeza and co-workers^199^ reported a transition of the measured chain-level dynamics, which were obtained from rheology experiments, from the Williams–Landel–Ferry (WLF) behavior to Arrhenius behavior in the temperature dependence of the frequency-scale shift factor (Figure 9B), when the glassy bridge is formed. The Arrhenius dependence is indicative of the (apparently noncooperative) activated exchange of adsorbed segments with bulk molecules, as opposed to the WLF relation of amorphous polymer chains. Concurrently, Sokolov and co-workers reported that substantial chain bridging among neighboring NPs promotes a frustration in chain packing that causes a reduction in mechanical properties.^201^

The formation of a percolating NP network has been reported based on mechanical property experiments^43,57,59,61,193^ or structural characterization (i.e., mass fractal structures) using X-ray/neutron scattering^164^ or transmission electron microscopy.^202^ However, much less is known about the dynamics of NP networks in PNCs due to the lack of experimental methods capable of providing direct dynamical information at the relevant length and time scales.

### 4.2 Collective NP Structures

To address the challenge, spherical SiO_2_ NPs with a mean particle diameter (*D*) of 18.2 or 23.6 nm were dispersed in a P2VP matrix with two molecular weights *M*_w_ = 9 and 38 kg/mol (we hereafter assign them as P2VP9k and P2VP38k, respectively). The size dispersity of these NPs was estimated to be about 18%. The NPs with *D* = 18.2 nm were utilized with the P2VP38k sample, while *D* = 23.6 nm was employed with the P2VP9k sample. A series of PNCs with increasing volume fraction of NPs (*ϕ =* 0.01, 0.06, 0.16, and 0.27*)* were prepared by means of dropcasting and subsequent solvent evaporation.^52^

As indicated by the rheology experiment, a gel point was identified at ϕ = 0.16, where *G*′ and *G*″ scale with an identical power law in frequency (see Figure 10A). It is hypothesized that the dynamics of the polymer bridging-induced network are primarily governed by the attachment-detachment process of adsorbed and confined polymers that facilitates the connection between the surfaces of two or more NPs.^203^ According to a previous phenomenological model analysis,^204^ Cheng and co-workers^53^ demonstrated that the shift factors of SiO_2_-filled PNCs from dielectric measurements follow:

16 $$a_{T}=a_{0}\left(T\right)\exp\left(\Delta E/\mathrm{RT}-\Delta E/RT_{\mathrm{ref}}\right)$$

where *T*_ref_ is the reference temperature and *a*_0_(*T*) is the dielectric shift factor which in the PNC was essentially equivalent to that in the pure polymer melt. Δ*E* is the activation energy for bridging polymer desorption per chain. Equation 16 was applied to the rheological data (indicated by the solid lines in Figure 10B), resulting in the following Δ*E* values for the P2VP9k and P2VP38k series: Δ*E* for the P2VP38k samples are approximately 60 and 100 kJ/mol at ϕ = 0.16 and ϕ = 0.27, respectively. Similarly, the Δ*E* values for the P2VP9k samples are approximately 50 and 10 kJ/mol at ϕ = 0.16 and ϕ = 0.27. It has been observed that that the magnitude of Δ*E* appears to exhibit a positive correlation with ID/2*R*_g_.

**Figure 10:**
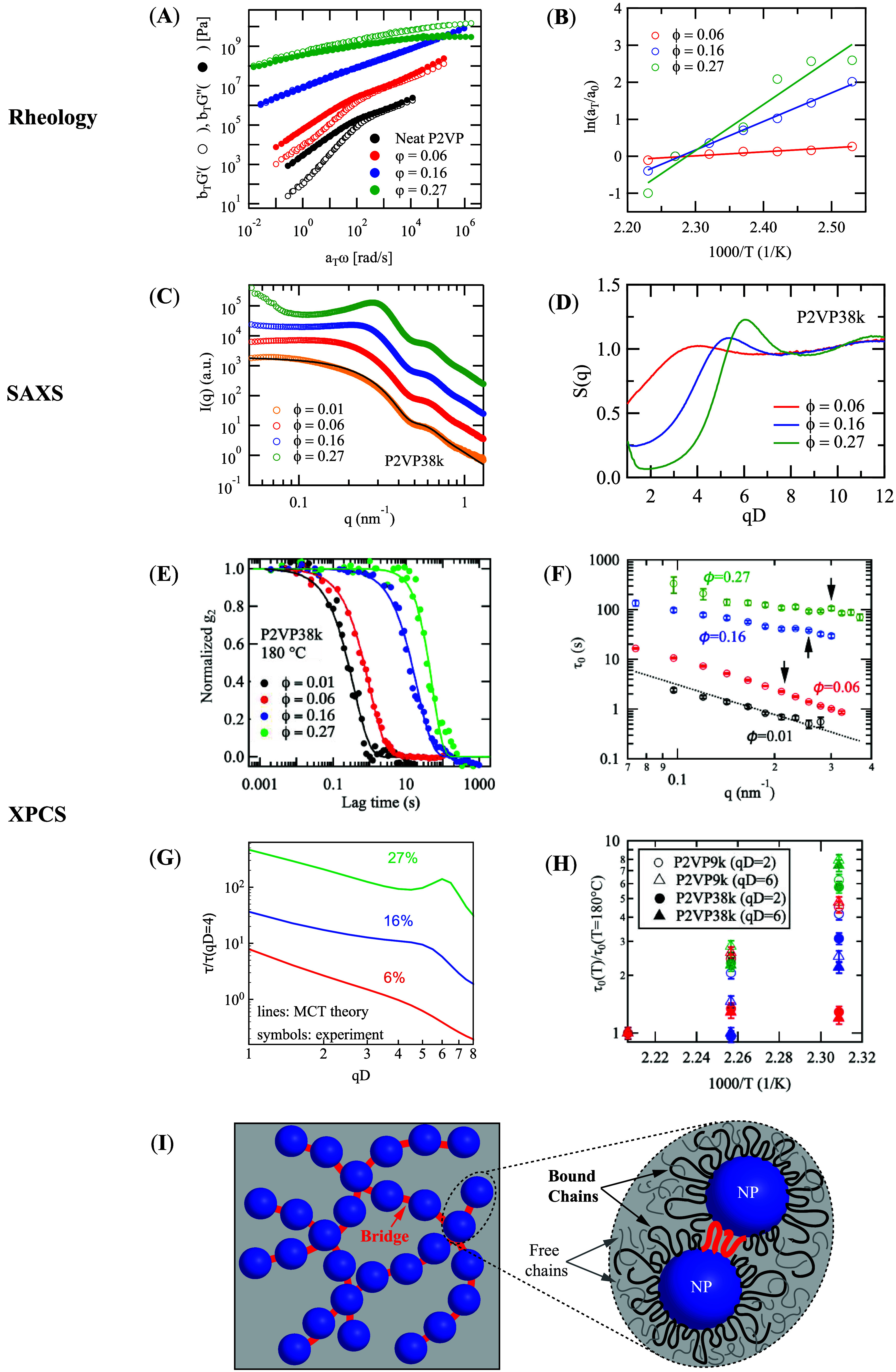
(A) Dynamic viscoelastic master curves *G*′(ω) and *G*″(ω) for the P2VP38k series generated by shifting small amplitude frequency sweeps according to time–temperature superposition (The reference temperature was set to 167 °C). *G*′(ω) and *G*″(ω) are vertically shifted by an additional shift factor *b*_T_ for clarity: *b*_T_ = 10, 100, and 1000 for ϕ = 0.06, 0.16, and 0.27, respectively. Time–temperature shift factors for the PNCs and the neat polymer used in the construction of dynamic master curves is shown in ref 52. (B) Shift factor (*a*_T_) for the P2VP38k series normalized by the shift factor for the neat P2VP38k melt (*a*_0_) as a function of inverse temperature. The solid lines show the best-fits of eq 16 to the PNC data to calculate the extra activation energy (Δ*E*). (C) SAXS intensity profiles *I*(*q*) (vertically shifted for clarity) for the P2VP38k series at 180 °C. The solid line corresponds to the best-fit to the data at ϕ = 0.01 on the basis of a polydisperse core–shell particle model. (D) Structure factor *S*(*q*) calculated according to SAXS intensities for P2VP38k at 180 °C. The *x*-axis is scaled with respect to the NP mean diameter (*D*). (E) Representative XPCS data for the P2VP38k samples at representative *q* = 0.23 nm^−1^ and 180 °C. The solid lines correspond to the best-fits of eq 11 to the data. γ = 1 for ϕ = 0.01, 0.06, and 0.16, while γ = 1.35 for ϕ = 0.27. (F) Relaxation time *τ vs*. *q* for the P2VP38k series at 180 °C. The dotted line shows the time scale associated with Stokes–Einstein limit of diffusivity. The arrows correspond to the primary (cage) peak position of *S*(*q*). (G) Collapsed *qD*-dependence of collective relaxation times. The τ values are normalized by the τ value at *qD* = 4. Vertical shifts (10 for ϕ = 0.16 and 100 for ϕ = 0.27, respectively) are used for clarity. Filled (P2VP38k) and open (P2VP9k) symbols represent the dataset for different MWs at 160 °C (square), 170 °C (circle), and 180 °C (triangle). Solid lines are theoretical predictions based on the MCT. (H) Temperature dependence of the normalized relaxation times at *qD* = 2 and *qD* = 6 for the P2VP9k and P2VP 38k series. The colors correspond to data collected at ϕ = 0.06 (green), ϕ = 0.16 (blue), and ϕ = 0.27 (red). (I) Cartoon of NP network structures at high loading ϕ > ϕ*_c_*. NPs are represented by blue circles connected by tight polymer bridges (represented by red lines). The right figure shows a cartoon of plausible chain structures near the polymer/NP interface. A few tails and/or loops composed of the BP adsorb on the surfaces of neighboring NPs concurrently, resulting in the formation of “doubly adsorbed” polymer segments (*i.e*., polymer bridges indicated in red). For entangled chains, the tails and (large) loops of the BP can entangle or interdigitate with free chains in a matrix. We caution that in a dense melt (vs. relatively dilute solution) polymer bridges do not consist of a single chain connecting two NPs. Rather, they represent a small piece of the polymer melt (multiple chains) that forms a thin cohesive structure that adsorbs on two NPs and spans the gap region of closest approach between them. Figures (A)–(I) are reproduced from ref 54.

The statistical NP microstructures are then evaluated from the SAXS intensity data *I­(q)*. Readers are encouraged to refer to review articles on how to extract size information about aggregates of NPs and their complex interactions in PNCs along with the reverse Monte Carlo analysis to provide a connection between the scattering data and real-space images.^205,206^ Representative SAXS profiles for the P2VP38k series at a temperature of 180 °C are presented in Figure 10C. The NP form factor *P*(*q*) (illustrated as the solid line in Figure 10C) was derived from *I*(*q*) for the dilute sample (ϕ = 0.01). The static structure factor of individual NPs at ϕ = 0.01 and the collective structure factor at ϕ = 0.06, 0.16, and 0.27 (hereafter referred to as *S­(q)* for both structure factors), were subsequently calculated by dividing *I­(q)* by the single NP form factor. As shown in Figure 10D, the results are depicted as a function of dimensionless wavevector *qD* for each *M*_W_ series of PNCs. The primary (cage) peak position *q* ^*^ of *S­(q)*, which is crudely related to an average ID, increases with increasing ϕ. This phenomenon is the anticipated trends of denser and more correlated local NP packing as NP loading grows. It should be noted that the magnitude of the ID value for the P2VP9k and P2VP38k series remains constant within the specified temperature range, all of which is above the bulk *T*_g_.^52^

Another important feature of *S*(*q*) is an upturn that emerges at *q* < q* (particularly at *qD* < 1). at ϕ = 0.16 and 0.27 for both series. In accordance with the polymer reference interaction site model (PRISM) integral equation theory,^52^ the upturn observed in the P2VP38K sample is attributed to long wavelength concentration fluctuations that occurs as a polymer-mediated network bridging type of phase transition is approached, as ϕ increases. The theory, with validated parameters, further provides a real space physical picture of the nanometer-scale microstructure: at ϕ = 0.16, the nearest neighbor coordination number of tightly bridged NPs is ≈ 2, suggesting the onset of a percolated network at this loading. A notable finding is the strong correlation between the theoretical predication and the rheology data for the P2VP38k sample (see Figure 10A). According to the PRISM theory, when ϕ is set to 0.27, the anticipated quantity of bridging nearest neighbors is approximately 4.^52^ This increase is both physically substantiated and qualitatively consistent with the large value of Δ*E* as deduced from the aforementioned rheology data.

### 4.3 Collective NP Dynamics

The collective NP dynamics in the P2VP matrix were investigated with *in situ* XPCS to measure the local motions of “markers”^104^ (i.e., SiO_2_ NPs which exhibit a high X-ray contrast relative to the polymer) over a wide range of *q* space (0.05 nm^–1^ < *q* < 0.4 nm^–1^) and time scales (10^–3^ s < *t* < 10^3^ s). It is important to note that, although these time scales can be regarded as “long” in comparison to those of the pure polymer melt at high temperatures, they may not be so in regard to the collective dynamics and self-diffusion of the NPs dissolved in the viscous polymer matrices. Therefore, the XPCS measurements are probing the relatively “short” time and length scale collective motions of NPs as influenced by the viscous friction associated with the vast majority of the polymer matrix and the constraints imposed by the small fraction of polymer segments that constitute long-lived bridges.

Figure 10E shows representative *g* ^(2)^ functions calculated from eq 9 at representative *q* = 0.23 nm^–1^ and 180 °C. Here, *g* ^(2)^ is normalized by β_0_ and the baseline for clarity (*g* ^(2)^ – 1/β_0_). The solid lines in the figure correspond to the best-fits of eq 11 to the data. For the P2VP9k and P2VP38k samples, γ = 1.35 ± 0.06 was observed for ϕ = 0.27, while γ = 1.00 ± 0.06 for ϕ = 0.01, 0.06, and 0.16. From the figure, the τ_0_ values for the P2VP38k samples exhibit an increase with ϕ. A similar trend was observed in the series of P2VP9k.^54^ Notably, at the highest NP loadings, 2 orders of magnitude increase in τ_0_ is evident compared to the baseline value at ϕ = 0.01. A comparable XPCS result was previously documented in SiO_2_ NPs embedded in entangled poly­(ethylene oxide) (PEO) melts.^207^

Figure 10F shows the *q* dependence of τ_0_ for the P2VP38k series at 180 °C. It is evident from the data that, for the dilute P2VP38k sample at ϕ = 0.01, τ_0_ ∼ *q* ^–*ε*^ with *ε* = 2, corresponding to a simple diffusive motion. The diffusion of NPs in a liquid is typically governed by the Stokes–Einstein (SE) relationship, which stipulate that the diffusion constant, denoted by *D*_SE_ = *k*_B_ *T*/(6π*R*_E_η) = 1/(τ_0_ *q* ^2^), where *k*_B_ is the Boltzmann constant, *T* represents the absolute temperature, and *R*_E_ signifies the effective radius of the particles, respectively. We then compared the experimental XPCS results to the modified SE relationship, under the assumption that the effective NP radius is affected by the BP, and that *R*_E_ = *R* + 1.1*R*_g_ = *R* + 0.0305 × *M*_*w*_^0.5^ (nm) for SiO_2_/P2VP PNC systems,^63^ and that η is the zero-shear viscosity of the PNC. The η value of the PNC was estimated by utilizing the following relationship^63^:

17 $$\eta =\eta_{0}\left(1+2.5\phi_{\mathrm{eff}}+6.2\phi_{\mathrm{eff}}^{2}\right)$$

where ϕ_eff_ is the effective NP volume fraction (ϕ_eff_ = ϕ­(*R*_E_/*R*)^3^) and η_0_ is the zero-shear viscosity of the P2VP bulk matrix. It should be noted that eq 17 is applicable within the context of dilute NP concentrations in PNCs. Colby and co-workers have reported that, for higher NP loadings of SiO_2_/P2VP PNCs, the coefficient of the third term is 14.1.^61^ As demonstrated in Figure 10F, the *q* dependence of τ_0_ for the P2VP38k 1% sample exhibits strong agreement with that predicted by the SE relationship, as indicated by the dotted line in Figure 10F. This finding is consistent with the previously validated SE relationship for a similar SiO_2_(*D* = 26 nm)/P2VP system at *ϕ =* 0.025 as determined by Rutherford backscattering spectrometry.^63^ Consequently, the diffusion detected by XPCS in the dilute NP limit tracks the self-diffusion of individual NPs (coated with the BP) dispersed in the viscous polymer matrix.

Conversely, Figure 10F demonstrates that the NP dynamics within the PNCs exhibit significant deviations from the self-diffusive behavior as the NP loading is increased. In addition, it was found that τ_0_ becomes less dependent on *q* at the higher loadings. In order to ascertain the existence of a “generic” *q*-dependence of the relaxation times between the two sets of PNCs, the reduced dimensionless wavevectors (i.e., *qD*) and the τ_0_ values normalized by τ_0_ at *qD* = 4 were utilized. The selection of *qD* = 4 is arbitrary. As summarized in Figure 10G, we can see that the data across all temperatures and *M*_W_ are collapsed onto a single master curve within the experimental uncertainties. This finding indicates that the behavior of τ_0_(*q*) is largely independent of polymer chain length, provided that the elementary ensemble-averaged polymer time scale in the PNCs is normalized out to zeroth order. This time scale is contingent upon both *T* and *M*_w_. Furthermore, the dynamical mode-coupling theory (MCT) was employed, incorporating the effect of bridging microstructure on the collective NP structures/dynamics over a wide range of length and time scales. A more comprehensive overview of the theoretical work has been summarized elsewhere.^54^ The integrated experimental-theoretical study has yielded two notable discoveries. First, the NP relaxation times are divided into the two regimes: short time self-diffusion dominant and collective network dynamics dominant. Second, the MCT analysis has been shown to make accurate predictions about the *q* and ϕ dependences of the NP relaxation times well for the unentangled PNCs. However, discrepancies have been indicated between theory and experiments emerge for entangled PNCs.

The study also involved an evolution of the effect of temperature on τ_0_ for the P2VP9k and P2VP38k series. Figure 10H provides a synopsis of the findings, with τ_0_(*q*) normalized by its value at 180 °C at *qD* = 2 and *qD* = 6. The figure indicates that the role of *qD* in the temperature dependence is negligible. Furthermore, for the P2VP9k series, the temperature dependence of the reduced τ_0_ appears to be insensitive to *ϕ.* Given that the viscosity of the neat P2VP9k and 38k melts decreases by approximately 4.4 and 9.2, respectively, when the temperature is increased from 160 to 180 °C, the temperature dependence of the NP dynamics tracks the viscosity change of a matrix polymer. This may be consistent with the results of the previous study,^52^ which demonstrated that the number of polymer bridges per NP increases with increasing NP loadings, leading to a more solid percolation. The motion of NPs connected by polymer bridges at ϕ = 0.16 and ϕ = 0.27 corresponds to an overdamped collective “vibration” motion of NPs in a polymer matrix, as illustrated schematically in Figure 10I. It is noteworthy that, in contrast to the P2VP9k series, where the ID/2*R*_g_ values are considerably larger, the P2VP38k series exhibits a diminished temperature dependence of the NP relaxation times, which is accompanied by an increase in ϕ. This phenomenon stands in direct opposition to the mechanical response of the PNCs.^54^ This opposing *T*-dependence suggests that the NP dynamics probed by XPCS may not be directly crucial for bulk PNC viscoelasticity. This deduction appears to be consistent with the key long-lived stresses arising from polymer bridges that mechanically connect NPs into a percolated network.

## 5 Structures and Dynamics of Polymer Networks

### 5.1 Background

A significant number of modern adhesives, sealants, coatings, and structural composites rely on the controlled transition from a liquid to a solid through the formation of a three-dimensional cross-linked polymer network, a process commonly referred to as curing.^208,209^ Many manufacturing processes in the electronics, automotive, and aerospace industries increasingly require precise control over curing under demanding process conditions. Because the microscopic structure and topology of the cross-linked network ultimately determine the mechanical, thermal, adhesive properties of thermosets,^210–215^ precise control over curing is essential. A distinguishing feature of thermosets is that their network topology can be systematically tailored through the cross-linking density.^216^ Achieving this level of control requires a molecular understanding of the coupled evolution of reaction kinetics, network topology, and polymer dynamics during curing.^217,218^ Because curing continuously modifies both the network topology and the underlying molecular dynamics, cross-linked thermosets provide an ideal model system for investigating hierarchical dynamic arrest across multiple length and time scales.

The primary challenge is the lack of characterization techniques that simultaneously provide the spatial and temporal resolution required to probe curing *in situ* under realistic processing conditions. A wide range of techniques — scattering techniques,^219–221^ confocal microscopy,^222^ NMR,^223,224^ image correlation microscopy,^225^ ultrasonic echography,^226^ BDS,^227^ DSC,^228^ and mechanical spectroscopy^229^ — have been employed to investigate gelation and vitrification in soft materials.

An ideal characterization technique should provide nanometer-scale spatial resolution because increasing evidence indicates that curing produces heterogeneous network domains with characteristic dimensions of several tens of nanometers.^212,213,230–233^ These domains originate from the heterogeneous growth of cross-linked fragments (microgels) during the pregelation stage, prior to the gel point (*t*_gel_) (Figure 11A).^234,235^ It is also expected that the bond formation probability via changes in temperature, reactivity, or catalyst concentrations affects the size distribution and morphology of microgels. These hierarchical structures play a critical role in determining the ultimate mechanical properties of thermosets.^236^ However, most state-of-the-art characterization techniques remain limited in their ability to perform *in operando* measurements under industrially relevant curing conditions. As a result, despite decades of investigation, the molecular mechanisms governing curing remains incompletely understood,^230,232,237^ and many industrial curing protocols continue to rely on empirical optimization through time-consuming trial-and-error approach.^231,238–244^ These limitations highlight the need for characterization techniques capable of simultaneously resolving the evolution of hierarchical structures and dynamics under realistic processing conditions, motivating the application of *in operando* XPCS.

**Figure 11:**
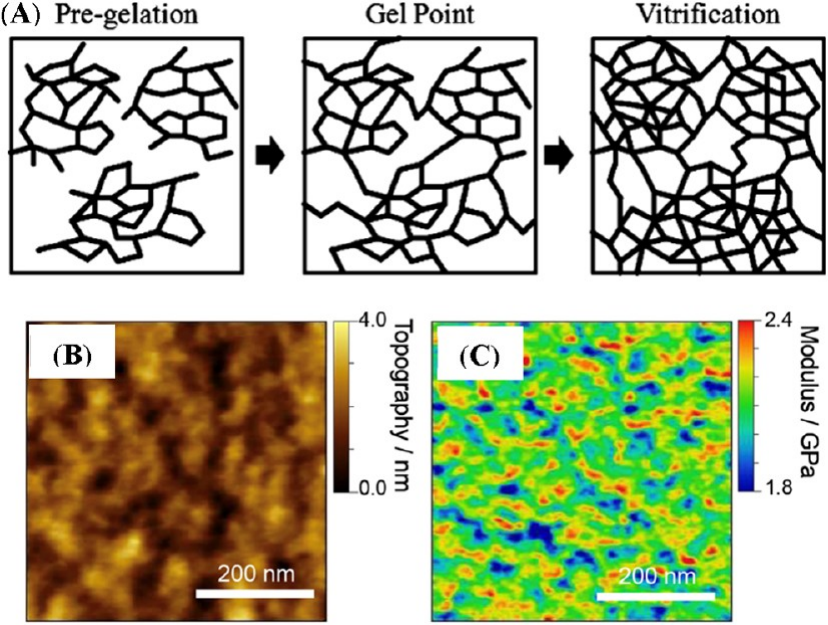
(A) Schematic representation of the formation of a heterogeneous network. The image is reproduced from ref 213. (B) Topography and (C) elastic modulus images of an epoxy composed of hydrogenated bisphenol A diglycidyl ether (HDGEBA) and 1,4-cyclohexanebis­(methylamine) (CBMA) with curing at 373 K for 0.5 h. The conversion degree was estimated to be 91% based on FTIR measurements. These images are reproduced from ref 248. It is noteworthy that the epoxy utilized in the study does not contain any NPs. The accompanying images serve to reflect the polymeric structure and the distribution of modulus values.

### 5.2 What We Know about Cross-Linked Polymer Network of Thermosets

Accumulating experimental and theoretical evidence indicates that network formation during curing is inherently heterogeneous rather than spatially uniform. The cross-linked network is composed of highly cross-linked nanoscale domains in an interstitial region of relatively lower cross-link density (see Figure 11A).^230,245–247^ The recent advent of atomic force microscopy-based infrared analysis^232^ or mechanical measurements^248^ evidenced the presence of nanoscale nodular structures associated with a network heterogeneity at the surface of epoxy systems (*ex situ*) (Figure 11B and C). It is noteworthy that the heterogeneous cross-linking structure on this scale is predicted to facilitate low-energy pathways through the resin, thereby providing a structural foundation for the relatively low fracture toughness of thermosets.^213,249^ This is a crucial aspect for the design and control of their mechanical properties. However, direct investigations on the growth of a network structure in thermosets are challenging due to the infusibility at high temperatures and insolubility in inorganic solvents.^250^ Two mechanistic pictures have been proposed to explain the origin of this heterogeneity. Morgan and Sahagan^213^ proposed that linear chains initially grow at a lower curing temperature until a low-density network expands over the system. Thereafter, unreacted group cross-links within the skeleton network (a homogeneous network) are formed. Conversely, the curing process at high temperatures leads to the formation of cross-linked domains, which subsequently interconnect, resulting in a heterogeneous network (Figure 11A). In contrast, recent MD simulations^233,250^ have suggested that once a reaction occurs, the temperature at the sites involved in the reaction is *locally* elevated, and cascade reactions near the sites are subsequently induced, developing the formation of a spatial heterogeneity regardless of macroscopic curing temperatures.

Synchrotron X-ray techniques, including SAXS and wide-angle X-ray scattering, have been identified as a promising approach for observing such behavior.^251–254^ Unlike conventional scattering methods, XPCS provides additional information related to the material dynamics across time scales. This allows for access to fast dynamics while still covering the time window for rheology and DSC measurements. Moreover, the recent development of *in operando* XPCS enables researchers to closely replicate industrial processing conditions, providing novel insights into the cross-linking and vitrification processes of cross-linkable polymers.^208,216,252,254–256^ Furthermore, XPCS is a microbeam scattering technique, with a typical beam diameter of tens of μm. It can probe materials in configurations as close to their real use case as possible (e.g., a 100 μm thin film sandwiched between two parts), while still providing spatial resolution within that layer.^217,256,257^ In previous work, a methodology for *in operando* XPCS for epoxy thermosets was developed, enabling the probing of the dynamics of a cross-linked polymer network using NPs embedded in the adhesive as “tracers” under relevant industrial processing conditions.^217,244,256–259^ Despite these advantages, the implementation of this approach is often constrained for industrial adhesives due to the following reasons: (i) high NP loadings, (ii) hierarchical structures of NPs including aggregates and agglomerates, and (iii) mixing of multiple NPs. Moreover, in the context of X-ray scattering measurements for polymer networks, the X-ray contrast between a network structure and a matrix composed of the same component is imperative.

As a representative example, we highlight our recent *in operando* XPCS study of a methyl methacrylate (MMA)-based structural adhesive without tracers.^260^ As summarized in Figure 12, the combination of XPCS, rheology, and DSC reveals the coupled evolution of structure and dynamics during curing and establishes quantitative structure–dynamics–property relationships. Network formation is not merely a structural process; rather, it involves the progressive coupling of evolving network topology and molecular mobility. The measurements reveal four distinct stages of curing: nucleation, the growth of polymerized MMA nanodomains, gelation, and vitrification (see Figure 12A). The following sections discuss the structural and dynamical characteristics associated with each stage of the curing process.

**Figure 12:**
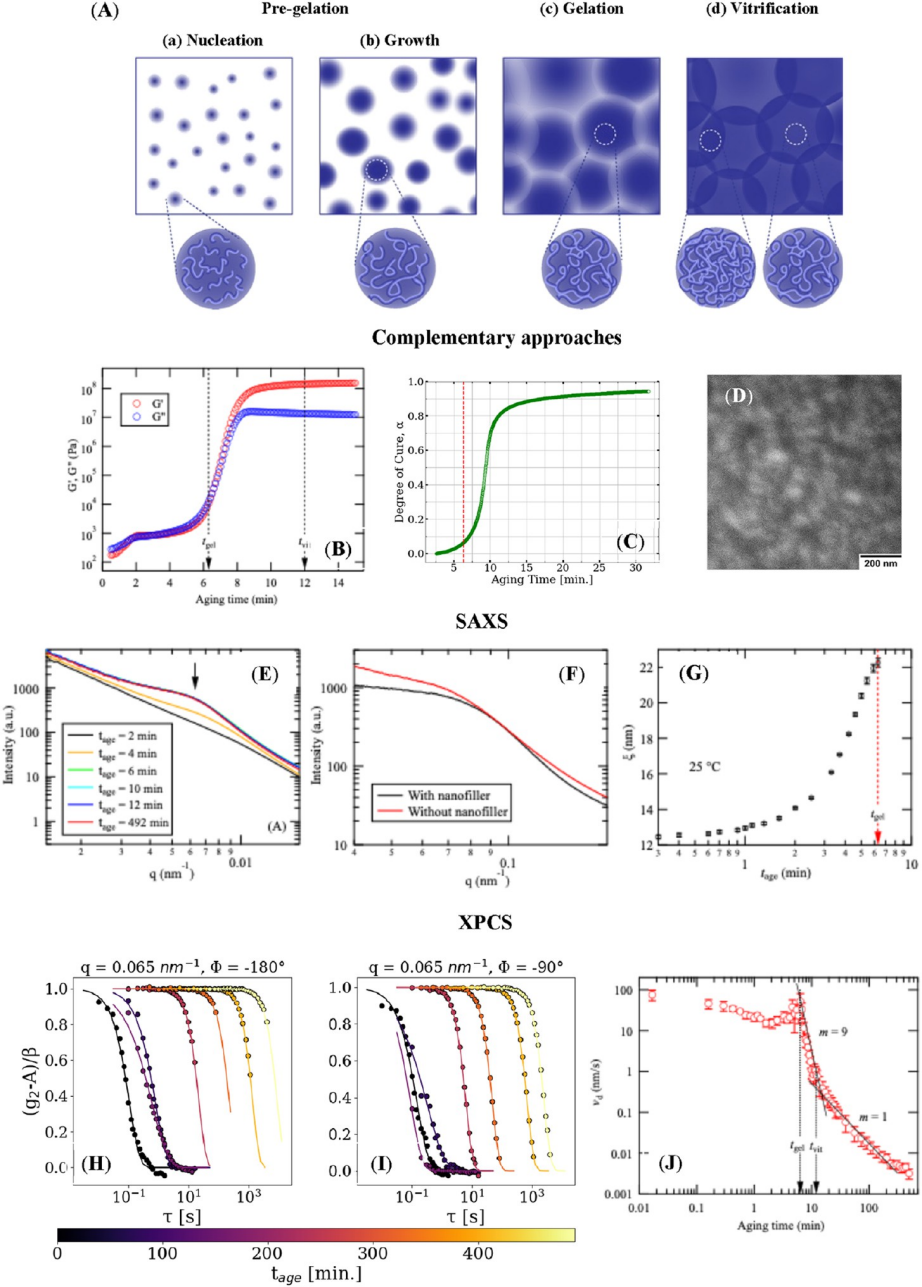
(A) Schematic of network growth in the acrylic adhesive during curing. In the pregelation stage at *t*_age_ < *t*_gel_, the polymerized MMA (PMMA) nanodomains are formed (stage a) and grow with *t*_age_ (stage b) in a liquid monomeric MMA phase. At *t*_age_ = *t*_gel_, the interconnection of the PMMA nanodomains occurs (stage c). The domain spacing of a network structure remains unchanged at *t*_age_ > *t*_gel_. As the reaction continues, vitrification occurs at *t*_age_ *= t*_vit_ (stage d), with *G*′ reaching a plateau value over *t*_age_. Based on the EFTEM images for the fully cured adhesive,^223^ it is expected that the boundary regions of the interconnected nanodomains are more cross-linked than the nanodomains themselves during the vitrification process. (B) Dynamic oscillatory shear rheology experiments on the acrylic adhesive during a cross-linking process. The storage modulus (*G*′) and loss modulus (*G*″) are shown as a function of aging time. The rheology data were collected at 25 °C using an oscillatory shear frequency of 1 Hz. The gel point and vitrification point are indicated by the dashed lines at *t*_gel_ = 6.3 min and at *t*_vit_ = 12 min, respectively. (C) Degree of cure of the acrylic adhesive cured isothermally at 25 °C. The red dashed line denotes the gel point at *t*_age_ = 6.3 min. (D) Bright field EFTEM image of the acrylic adhesive after it has undergone a 24 h curing process at 25 °C. The dark phase of the image corresponds to the regions of higher cross-linking (i.e., higher density). In contrast, the bright phase corresponds to the regions of less cross-linking (i.e., lower density). (E) Representative SAXS curves of the acrylic adhesive cured at 25 °C at *t*_age_ < *t*_gel_, *t*_age_ ≈ *t*_gel_, *t*_age_ ≈ *t*_vit_, and *t*_age_ > *t*_vit_. The correlation peak is identified by the arrow. (F) SAXS curves of the acrylic adhesive cured with and without NPs at *t*_age_ = 9 min at 25 °C. (G) *t*_age_ dependence of the characteristic length (ξ) at 25 °C. The red vertical line denotes the gel point, at *t*_gel_ = 6.3 min. Fits of the normalized one-time autocorrelation functions to the KWW stretched exponential equation for the acrylic adhesive without NPs cured at 25 °C at *q* = 0.065 nm^–1^, (H) parallel to (Φ = −180 °) and (I) perpendicular to (Φ = −90 °) the substrate surface, and at a variety of *t*_age_. (J) Displacement velocity for the acrylic adhesive at 25 °C determined via XPCS. The solid lines show power law functions overlaid on the data as guides for the eye. The power law exponents are labeled with “*m*”. The dashed lines indicate the gel point and vitrification point determined from the rheology data. Figures (A)–(J) are reproduced from ref 260.

### 5.3 *In Operando* XPCS

#### 5.3.1 Hierarchical Structures of Cross-Linked Polymer Network

The acrylic adhesive was an industrial formulation of a two-component (LOCTITE HHD 8540) supplied by Henkel Corporation. The predominant component (henceforth designated as “Component A”) was primarily composed of MMA monomers. The minority component (hereafter referred to as “Component B”) contained dibenzoyl peroxide.^261^ The Components A and B were mixed in a 10:1 ratio, employing a static mixer to initiate the free-radical polymerization of the MMA monomers. The acrylic adhesive was formulated with a small quantity (a mass fraction of 0.8%) of Aerosil 200 fumed SiO_2_ NPs (Evonik) as a viscosity modifier. Concurrently, a two-component acrylic adhesive without SiO_2_ was also formulated (henceforth designated as the “acrylic adhesive without NPs”) for XPCS measurements. A custom positioning stage for *in operando* X-ray studies was designed to mimic an industrial assembly process (including extrusion, deposition, and cross-linking).^260^ The experiments were set up such that the start of data collection coincided with the mixing of the acrylic adhesive components.

As shown in Figure 12B, the time dependence of *G*′ and *G*″ for the acrylic adhesive during the curing process at 25 °C with an oscillatory shear frequency of 1 Hz is demonstrated. The gel point (*t*_gel_) was determined to be *t*_gel_ = 6.3 ± 0.1 min independent of the oscillation frequency. After about 12 min, when the degree of cure (α) was about 0.85, as determined by the DSC experiment (Figure 12C), *G*′ reached a plateau value. This finding indicated a substantial decrease in the cross-linking reaction rate.^262^ The phenomenon is referred to as the onset of vitrification (*t*_vit_), as previously reported.^263^

Figure 12D presents a zero-loss filtered bright-field energy filtered TEM image of the acrylic adhesive sample following a 24-h curing period at 25 °C, corresponding to α = 1.0. The measurement was conducted at room temperature (≈25 °C) with an average electron density of approximately 50 e^–^/Å^2^. As seen in Figure 12D, the dark regions exhibit slightly higher mass density compared to the bright regions. This indicates that the dark regions undergo more active reactions in comparison with the bright regions.^233^ Consequently, the image corroborates the hypothesis concerning network structures at the nanoscale within the acrylic adhesive. The average separation distance between the bright regions is estimated to be about 100 nm, a value that aligns with the SAXS results, which will be discussed subsequently.

Figure 12E shows the *in situ* SAXS curves of the acrylic adhesive that was cured at 25 °C as a function of curing time, *t*_age_. As the curing time progresses, a broad peak emerges at q* ≈ 0.075 nm^–1^, which corresponds to a size of approximately 100 nm. This development is observed at *t*_age_ ≈ 6 min. In order to discuss the origin of this peak, we studied the time-resolved SAXS results for the acrylic adhesive without NPs. As demonstrated in Figure 12F, the peak also emerged in the acrylic adhesive without NPs, thereby substantiating that the observed structure development emanated from the polymer matrix. The observed discrepancy in the scattering intensity between the samples containing and those devoid of SiO_2_ NPs is attributable to the scattering from the aggregation of the SiO_2_, which exceeds a few hundred nm. In conjunction with the TEM image displayed in Figure 12D, it is reasonable to conclude that this scattering peak is attributed to the spacing of neighboring nanodomains. The domain spacing, which is calculated by 2π/q*, remains constant over the duration of the experiments, which extends up to *t*_age_ = 500 min. The invariant (Q*) calculated from the SAXS data remains constant at *t*_age_ < 1 min following the mixing process.^260^ Thereafter, an abrupt increase was observed, which subsequently reaching a plateau at *t*_age_ = *t*_gel_ = 6.3 min. Consequently, it can be deduced that the microscopic structure that emerges as a result of the curing process and is probed by SAXS attains stability following the gel point.

In the following section, we address the structural evolution prior to the gel point. The Debye–Bueche (DB) equation,^264^ which is based on the assumption of a random two-phase structure, was employed to analyze the scattering data at *t*_age_ < *t*_gel_ for the acrylic adhesive:

18 $$I\left(q\right)=\frac{C}{{\left(1+\xi^{2}q^{2}\right)}^{2}}$$

where ξ is the characteristic length, designated as the DB correlation distance, and is equal to half of the “chord length (or the harmonic mean of mean transversal length) of each phase”,^265^ and *C* is a numerical constant. As shown in Figure 12G, the parameter ξ is presented as a function of *t*_age_. The existence of two different regimes at *t*_age_ < *t*_gel_ is evident: (i) ξ remains almost constant at *t*_age_ ≤ 1 min when the adhesive is in its liquid phase (see Figure 12B). It is hypothesized that the MMA monomers polymerize into poly­(methyl methacrylate) (PMMA) and form the PMMA nanodomains in the MMA matrix. (ii) At *t*_age_ > 1 min, the nanodomains start to grow rapidly and the characteristic length reaches a final value (ξ ≈ 22 nm) at *t*_gel_. The nanodomain structures bear a resemblance to cross-linked network fragments, also known as “microgels”,^212,213,230–233^ prior to the gel point.^234,235^ However, the evidence is not conclusive regarding the cross-linking of PMMA chains within the nanodomains, as the X-ray contrast (i.e., electron density) can also be attributed to the density difference between MMA and PMMA.

#### 5.3.2 Hierarchical Dynamics of Cross-Linked Polymer Networks

Figure 12H and I show representative one-time correlation (*g* ^(2)^) functions for the acrylic adhesive calculated using eq 9. The parallel configuration, defined by the azimuthal angle (*Φ)* of −180°, relative to the substrate surface, and in the perpendicular configuration (Φ = −90°) are shown here. The solid lines correspond to the best fits of the KWW equation (eq 11) to the data, thereby obtaining the relaxation rate, Γ = 1/τ_0_ (s^–1^), as a function of *q.* The detailed XPCS analysis protocol for calculating the *g* ^(2)^ function from the two-time correlation functions and other parameters describing the characteristic dynamics of the cross-linking reaction have been described elsewhere.^256,257^ The dynamics of the acrylic adhesive in the absence of SiO_2_ NPs are in good agreement with those of the acrylic adhesive with NPs. This ensures that the XPCS experiments conducted effectively track the dynamics of the polymer structures within the acrylic adhesive itself rather than those of the SiO_2_ NPs present in the acrylic adhesive.

In the context of soft matter systems probed by XPCS, the relationship between Γ and *q* is a subject of particular interest, as it serves as an indicator of whether the observed dynamics are diffusive or ballistic.^266,267^ The findings indicated a linear relationship between Γ and *q* (Γ ∝ *q*) before and after the gelation for all values of *q* and Φ. A similar relationship has been observed for colloidal gels, concentrated emulsions, and NPs suspended in polymer matrices.^267–270^ The slope between Γ and *q* represents the displacement velocity (where Γ = *v*_*d*_ *q*),^268,269^ which quantifies the local displacement of the isolated or interconnected nanodomains.

As shown in Figure 12J, the *v*_d_ values for the acrylic adhesive are presented as a function of *t*_age_ at 25 °C. It was ascertained that the displacement velocity remains constant for the acrylic adhesive, both with and without the incorporation of SiO_2_ NPs. This finding serves to substantiate the efficacy of our XPCS experiments in detecting the dynamics of the isolated or interconnected PMMA nanodomains within the acrylic adhesive. The displacement velocity at *t*_age_ < 2 min, corresponding to the dynamics of the isolated PMMA nanodomain in the MMA matrix, decreased from ≈80 to ≈15 nm/s. Given the observation that the sizes of the PMMA nanodomains remain constant over time,^260^ this substantial decrease can be interpreted as an increase in the viscosity of the MMA matrix due to the polymerization as the reaction progresses. At 2 min < *t*_age_ < 6.3 min, a modest maximum in *v*_d_ is evident. This phenomenon is expected to be associated with the exothermic process of the acrylic adhesive.^260^

The question that must be addressed is the nature of the dynamics subsequent to the onset of gelation. As mentioned above, the growth of the individual PMMA nanodomains is completed at *t*_age_ > *t*_gel_. In contrast, a marked slowing down of the nanodomain dynamics becomes evident at *t*_age_ > *t*_gel_. This indicates that the interconnection of the PMMA nanodomains occurs at *t*_age_ = *t*_gel_. Consequently, the displacement velocity decreased by approximately 2 orders of magnitude at *t*_gel_ < *t*_age_ < *t*_vit_ (≈ 12 min). As demonstrated in Figure 12B and C, the degree of cure (α) exhibited a significant increase during this time period, leading to abrupt changes in the macroscopic properties. Therefore, it can be concluded that the significant suppression of *v*_d_ is attributed to the growth of the network structure (i.e., the interconnection of the PMMA nanodomains) that spans the entire sample.

As time further progresses, the cross-linking reaction slows down substantially. Concurrently, the adhesive undergoes a transition to the vitrified state, as evidenced by the absence of significant data in both the DSC and rheology analyses. This transition marks the adhesive’s progress to a fully developed solid state. In contrast, the XPCS results clearly demonstrate the further slowing down of the network dynamics associated with the vitrification process. As shown in Figure 12I, the XPCS results indicate a complete decay of one-time correlation functions even at *t*_age_ ≈ 500 min. Moreover, this decay exhibits a rate that exceeds that of exponential relaxation, otherwise known as compressed decay. A similar set of characteristics has been observed in fractal colloidal gels formed by the aggregation of polystyrene colloids^268^ and concentrated hard-sphere colloidal suspensions.^271^ Furthermore, a gradual yet continuous decrease in the *v*_d_ values up to *t*_age_ ≈ 300 min is evident. When the data is plotted on a log–log scale (see Figure 12J), the postgelation dynamics appear to follow a two-step power-law decay (i.e., *v*_d_ ∝ *t*_age_^–*m*^, where *m* is the power-law decay exponent). The power-law exponent is estimated to be *m* ≈ 9 for *t*_gel_ < *t*_age_ < *t*_vit_ and decreases to *m* ≈ 1 for *t*_age_ > 30 min. A crossover region has been identified between *t*_vit_ < *t*_age_ < 30 min. The *v*_d_ displays a plateau value of 0.05 nm/s at *t*_age_ > 300 min.

The “long-tailed” vitrification process is evident in the acrylic adhesive, a phenomenon that is also observed in other industrial adhesives, such as one-component heat-curable epoxy resin (LOCTITE 3536 CSP, Henkel Co.)^217^ and dual-cure (thermal + UV light) two-component (Acrylate/Epoxy, LOCTITE VP 10997-085, Henkel Co.)^257^ adhesives. Of particular significance is the observation that the long-tailed vitrification process exerts a direct influence on the ultimate mechanical property of adhesives.^257^ Consequently, the capacity to resolve the final stage of cure for a “macroscopic” solid constitutes one of the paramount benefits of conducting *in operando* XPCS experiments. Additionally, it was determined that the overall dynamics during the curing process of the acrylic adhesive are nearly independent of the selected curing temperatures up to 80 °C. This suggests that the dynamics of polymerization, gelation, and vitrification of the acrylic adhesive are not affected by thermal effects. In the event that the cure temperature is excessively high, thermosets will degrade without undergoing vitrification. Conversely, if the cure temperature is insufficiently low, thermosets will vitrify without gelation.^272^ Therefore, it is imperative to optimize the cure temperature to achieve the desired final strength of the thermosets, while considering that curing at “room temperature” (approximately ranging from 20 to 40 °C range, contingent upon the prevailing ambient climate conditions) is preferable for the customer. Consequently, a characterization technique capable of reliably probing these dynamics is undoubtedly useful in controlling the end-use properties.

Epoxy vitrification is one of the best-studied examples of chemical vitrification and has long served as a model system for elucidating the relationship between polymerization, network formation, and glass formation. A major advance over the past three decades has been the recognition that no single experimental technique can fully capture the complexity of vitrification. Instead, the integration of complementary techniques—including DSC, dynamic mechanical analysis, rheology, Fourier transform infrared spectroscopy, and BDS—have provided a comprehensive picture by probing distinct but interconnected aspects of the curing process.^227,228,273–275^ Collectively, these studies establish vitrification as a multiscale chemo-mechanical phenomenon governed by the interplay between chemical conversion, network development, and progressive slowing of molecular dynamics. However, despite their complementary strengths, these techniques provided limited spatial resolution and therefore cannot directly resolve how network heterogeneity evolves on nanoscale length scales during vitrification. The integration of X-ray and neutron scattering techniques offers a pathway to bridge this gap by simultaneously probing structural evolution and dynamics. This integrated experimental framework provides a valuable blueprint for investigating vitrification in PNCs, where the challenge is even greater because dynamic arrest is further coupled to spatial heterogeneity introduced by NPs.

At the molecular level, vitrification occurs when the structural relaxation time increases dramatically upon approaching *T*_g_. This pronounced slowing of molecular dynamics is associated with the growth of cooperative rearranging regions, in which increasingly larger groups of polymer segments undergo collective motions.^276^ In PNCs, NPs perturb these cooperative motions by creating polymer–NP interfacial regions whose dynamics differ from those of the bulk polymer.^227^ Consequently, the vitrification process depends strongly on the polymer–NP interactions.^38,199^ Owing to the heterogeneity of environments, verification in PNCs is not a uniform transition, but rather occurs over a broad range of temperatures and relaxation times. Moreover, NPs influence hierarchical dynamics spanning multiple length and time scale, including segmental dynamics, Rouse dynamics, entanglement dynamics, long-range diffusion, network relaxation in cross-linked polymers, and the relaxation of NP network. Consequently, vitrification in PNCs should be regarded as a multiscale dynamic arrest process that couples molecular motions across different hierarchical levels. This modern perspective has become pivotal in comprehending the mechanical reinforcement, durability, and long-term performance of PNCs, particularly SiO_2_- and CB-filled elastomers.

## 6 Outlook and Prospects

Throughout this Perspective, we have shown that the synergistic integration of X-ray and neutron scattering, in conjunction with MD simulations, provides a comprehensive unprecedented view of the hierarchical structures and dynamics of polymers and NPs. By combining complementary experimental and computational approaches across multiple length and time scales, these methodologies establish quantitative hierarchical structure–dynamics–property relationships that provide a molecular foundation for the rational design of advanced PNCs. The high-resolution structural and dynamical information obtained from these approaches reveals the complex physical mechanisms governing reinforcement and provides molecular design principles for next-generation hybrid PNC materials necessary for future applications both within and outside academia.

The impact of this integrated strategy is illustrated by its successful application to the molecular design of SiO_2_-filled tire compounds developed by Sumitomo Rubber Industries, Ltd. (SRI). By integrating NBS with MD simulations, the SRI team identified the mobility of BP chains grafted through SCAs as a critical molecular parameter governing local fatigue damage at the elastomer–SiO_2_ interface. The resulting microscopic fatigue damage subsequently propagates throughout the material, ultimately leading to macroscopic fracture. Optimizing the molecular length of the SCA resulted in a significant improvement in abrasion resistance without compromising rolling resistance or wet grip, culminating in the commercialization of the ENASAVE NEXT II tire in 2016. This example illustrates how quantitative structure–dynamics–property relationships can transform the design of PNCs from empirical optimization to molecularly informed materials engineering. Together, these integrated methodologies establish a pathway from molecular characterization to predictive modeling and rational materials design.

The following sections discuss emerging opportunities for polymer nanocomposite research enabled by advanced X-ray and neutron scattering techniques, MD simulations, and machine learning.

### 6.1 QENS

The power of neutron scattering lies in the sensitivity to both coherent and incoherent dynamic structure factors. These govern the distinct and self-part of the van Hove correlation function (eq 6). Recent trends in QENS measurements involve the separation of coherent and incoherent dynamic structure factors through the use of neutron polarization analysis (NPA).^277–279^ The most successful instrument in this regard is arguably the polarized LET instrument at ISIS, UK,^277^ which is a TOF-QENS instrument and has the capacity to measure dynamics from 10s ps to 100s ps.^280,281^ However, the time domain is too short for PNC studies. Presently, there is a vision for the expansion of dynamic ranges to encompass longer time scales, and there is ongoing discourse regarding the development of NBS in conjunction with NPA (see, for instance, ref 282). The fundamental principles underlying the intensity modulated NSE technique suggest its capacity to differentiate between coherent and incoherent scattering components.^283^ These efforts will provide more accurate comparisons to computer simulation studies, a future direction that neutron scattering will experimentally provide purely self- and distinct part of dynamic structure factors.

The self-part of the Rouse dynamics has been studied using NBS at the range of *q* where incoherent scattering predominates. Recent advancements in NSE technology have led to a significant expansion in its capacity to encompass a broader range of space domains, while concurrently enhancing its counting efficiency. The development of the WASP instrument at ILL^284^ has enabled the NSE measurement at wide scattering angles, analogous to those covered by NBS. A recent example in a highly cross-linked PB system with a combination of NBS and WASP successfully separated coherent and incoherent contributions to the scattering functions. These techniques measure the coherent and incoherent components using different forms of linear combinations in NBS and NSE. This facilitated a more detailed discussion about cross-linking dynamics at the segmental level.^159^ This type of approach will lead to an increase in the ability to understand and analyze complex hierarchical dynamics in PNCs, while experimental efforts will be more demanding. It is also noted that the machine learning approach for NSE data reduction has been developed to gain better data quality. Such an approach may help faster data acquisition in future experiments.^285^

The global capacity to perform QENS experiments is constrained by two factors: the number of reasonably large neutron facilities and the number of instruments available to users. The scarcity of NSE spectrometers is particularly pronounced, with a limited number available for use, particularly in cases requiring access to longer Fourier times that extend beyond a few hundred nanoseconds to probe the local reptation dynamics of polymers.^76^ In addition to the improvements currently being implemented in existing spectrometers, the development of new neutron facilities, such as the European Spallation Source and the Second Target Station at the Spallation Neutron Source, will result in the addition of QENS capabilities. The increased number of instruments, as well as their improved performance, will lead to enhanced measurement efficiency, with the capacity to span time scales ranging from subps to μs and length scales ranging from angstroms to some 10 nm in the future. It is noteworthy that the development of multiple accelerator-based compact neutron sources (with comparatively lower flux) is currently underway or in the planning stages on a global scale. The integration of large-scale neutron facilities with laboratory instruments, including as BDS and NMR, has the potential to enhance our understanding of hierarchical polymer dynamics in PNCs, given that it involves only a small amount of sample and no isotopic labeling requirement.

### 6.2 Emerging Quasielastic Scattering Techniques

While selective deuteration is essential to provide weight to coherent and incoherent scattering contributions in neutron scattering, X-ray scattering inherently measures coherent scattering. The development of resonant γ-ray scattering technologies in the field of polymers would create a unique opportunity to measure the collective dynamics of polymer chains through quasielastic γ-ray spectroscopy (QEGS). The initial effort to quantify the dynamics of polydimethylsiloxane chains in a diluted benzene solution using QEGS was reported in 1990.^286^ This effort demonstrated an extension of the energy resolution to several μeV in the X-ray-based technique. Recent advancements using multiline frequency comblike resolution function have facilitated the measurement using a multiline frequency Mössbauer absorption spectrometer (MLS), which are designated as MLS-QEGS. This approach, developed by Saito and colleagues,^287^ has led to significant improvements in both the intensity and the energy resolutions, extending the range from several μeV to 10s neV. Consequently, this approach encompasses polymer chain correlation dynamics within the time span from 100 ps to 10s ns.

Multiline resonant γ-rays have also been utilized in a time domain interferometry (TDI-QEGS), which is capable of measuring the dynamics of various materials within the range of several ns to 100s ns.^288–290^ The initial implementation of this technique in the context of polymer dynamics research was to assess decoupling mechanisms for α- and slow β-relaxation processes in proximity to the *T*_g_ of hPB. In comparison to the preceding NSE investigations conducted by Richter and colleagues,^170,291,292^ the TDI-QEGS study demonstrated that the α-process exhibits predominance below and at the first structure factor peak, while the β-process manifests solely at higher *q* values above the structure factor peak below the transition temperature.^293^ The impact of SiO_2_ NPs on the α-relaxation in hPB,^294^ as well as strain-dependent cross-linked PB for α- and β-relaxations,^295,296^ was measured using the TDI-QEGS. The results indicated that the technique is useful for studying PNCs without the need for deuteration of polymeric samples.

In conjunction with inelastic X-ray scattering (IXS), conventional NBS and NSE, the spatial and temporal coverage of these spectrometers is summarized in Figure 2. The length and time scales are commensurate with sound waves and phonon propagation,^297^ the polymer side chain motions, the Rouse dynamics, the local reptation dynamics, and the NP diffusion dynamics. In the context of PNCs, confinement effects have been shown to significantly alter the long-time scale dynamics.^15^ This phenomenon can be captured using XPCS, TDI-QEGS, and NSE, which are derived from the distinct parts of the dynamic structure factors. Segmental motions and the self-part of Rouse dynamics, as well as side chain motions, are measured using QENS and WASP-type NSE. Further local distinct dynamics such as α- and β-relaxations of polymer chains, as well as higher-order correlations, such as cross-linker correlation dynamics, is achieved by combining TDI- and MLS-QEGS, QENS, and NSE. The combination of these techniques will elucidate the hierarchy of steady-state dynamics from the ps to μs time scale. This will facilitate understanding of the intricate interplay among polymers, additives and NPs in PNC studies.

### 6.3 XPCS

The potential applications of XPCS analysis extend beyond the extraction of time-scales and compression exponents via the KWW form (see eq 11). When samples are subjected to external forces, such as shear,^298,299^ the functional form of *g* ^(2)^ becomes more complex than eq 11. A unified description based on the transport coefficient of the system has been proposed.^300^ Inverse transformations have also been employed to unravel multimodal dynamics in materials.^255^ In general, XPCS is employed to measure translational diffusion, while an analysis of angular-temporal cross-correlations is required to measure rotational diffusion.^301^

The *C* (*q*, *t*_1_, *t*_2_) function (see eq 10) can also be used to track the evolution of a given state of the sample’s electron density distribution over time. In the context of a sample undergoing a cyclic external deformation (e.g., unilateral strain or shear), *C* (*q*,*t*_1_ = const., *t*_2_ ≥ *t*_1_) shows periodic “echoes” of correlations, provided that the deformation remains reversible (and any intrinsic diffusive NP dynamics possesses time scales that are significantly longer than the measurement duration). The observed decay of these echo amplitudes is indicative of structural yielding, a phenomenon that can occur at the microscale well before the detection in macroscopic measurements.^302^ Speckle tracking^303^ has been demonstrated to quantitatively measure strain even in completely amorphous materials.

With the exception of speckle visibility spectroscopy,^304,305^ which has limitations for heterogeneous and out-of-equilibrium samples, XPCS requires the collection of a time-series of scattering patterns from the same scattering volume of the sample. It is imperative to exercise caution during data acquisition, as beam damage effects may manifest not only in conjunction with accumulated X-ray dose, but also as a function of dose rate, independent of the effects of dose accumulation. To manage the X-ray dose, the beam can be attenuated (at the expense of the signal-to-noise ratio of the correlation function) or spread over a larger scattering volume by increasing the beam size (at the expense of spatial resolution and potentially speckle contrast).

The capacity to resolve speckles while illuminating samples with beams measuring tenths of micrometer-sized beams has enabled XPCS studies of soft matter systems’ dynamics without the utilization of “tracer particles”.^260,306^ The denoising of *C* (*q*, *t*_1_, *t*_2_) functions by machine learning methods^307,308^ has been demonstrated to further increase the signal-to-noise ratio, thereby improving the time resolution or reducing the required X-ray dose/rate in XPCS measurements.

The time-resolved partially coherent scattering data set that constitutes the raw data, which serves as the fundamental data set for XPCS, possesses not only the aforementioned wealth of information related to the sample’s dynamics but also all the structural information that can be extracted from conventional, incoherent scattering. Furthermore, the employment of microfocused X-ray beams enables the discernment of spatial heterogeneities within a sample. This facilitates the acquisition of images that elucidate the structural and dynamic characteristics, in addition to their time evolution.^256,257,309–311^

As demonstrated in Sections 4.3. and 5.3, the integration of time-resolved coherent scattering with *in situ*/*in operando* experiments establishes a robust multimodal experimental framework. Consequently, while XPCS was previously the domain of specialized beamlines (e.g., ID10 (ESRF), 8ID (APS), P10 (Petra-III), and CHX (NSLS-II)), an increasing number of existing and planned SAXS/WAXS beamlines, such as ID02 (ESRF), CoSAXS (MAX-IV), CATERETÊ (Sirius), and AMP (NSLS-II), are capable of employing coherent scattering techniques to obtain information beyond the sample’s average microstructure. Furthermore, the enhancement in brightness of fourth-generation synchrotron sources, in conjunction with innovative time-stamping detectors, has created a new paradigm for the measurement of polymer dynamics in PNCs within the microsecond and submicrosecond time window.^312^ This advancement signifies a substantial progress in the field, bridging the existing gap between the NSE and XPCS time domains, as illustrated in Figure 2.

### 6.4 MD Simulations

Simulations have become central to interpreting neutron and X-ray scattering data and to elucidating the hierarchical dynamics that govern PNCs. Their capacity to calculate neutron-observable quantities, including intermediate scattering functions, interfacial density profiles, and relaxation spectra, while simultaneously resolving chain conformations and interfacial architectures that are inaccessible to experimental methods, renders them indispensable for establishing a connection between molecular-scale processes and mesoscale mechanical behavior.

AMD simulations provide the most direct link to neutron scattering because *S*(*q*,*t*) can be computed from atomic trajectories using the van Hove correlation formalism, a methodology established in foundational neutron–simulation work on polymer dynamics.^313–316^ While AMD simulations demonstrate proficiency in capturing local chemistry, the nanometer and nanosecond scales probed by NSE and NBS frequently surpass the tractable limits of AMD. CGMD addresses this by enabling larger system sizes and longer trajectories. Furthermore, CGMD unveils features that are inaccessible through scattering methods, such as interpenetration depth, density gradients, entanglement dilution, and NP bridging networks. At ultrahigh NP loadings, polymer bridges have been shown to suppress particle rearrangements and enhance stiffness.^317^ Generic CG models similarly reproduce size-dependent reinforcement and adhesion-controlled mechanical trends across NP types, thereby confirming the dominant role of interface topology and chain connectivity.^318^

XPCS provides a complementary perspective on larger-scale and slower dynamics involving NP networks, NP diffusion, and mesoscale viscoelastic relaxation. Despite the challenges associated with direct molecular simulation of XPCS observables, modern machine-learning (ML) inference techniques have paved the way for reliable interpretation of sparse or noisy time-dependent scattering data. Gaussian-process reconstruction (GPR) has been demonstrated to significantly improve counting statistics and reveal universal convergence behavior in time-resolved SANS.^319^ Bayesian inference has been shown to reconstruct anisotropic 2D scattering patterns from incomplete measurements,^320^ and neural-network inversion has been demonstrated to extract polymer or colloidal interaction parameters directly from structure factors.^321,322^ Related 2D ML-enabled frameworks, such as CREASE-2D, which employs surrogate forward models and optimization strategies to interpret full 2D SAXS/SANS patterns,^323^ has demonstrated how anisotropy-preserving, data-driven approaches can complement GPR- and Bayesian-based neutron inference and provide mesoscale structural descriptors relevant to XPCS. Collectively, these ML surrogates establish scalable routes for connecting simulation outputs, local dynamics, mesoscale correlations, and relaxation spectra, to experimentally measured XPCS autocorrelation functions.

Machine-learned interatomic potentials (MLIPs) broaden the accessible simulation regime by providing near-DFT accuracy at scale for chemically complex polymer–NP interfaces. It has been demonstrated that foundation-level architectures, including Orb-v3, AIMNet2, and MACE-OFF, offer transferable predictions of polarization, environment-dependent charge regulation, and multielement interactions. These architectures also maintain computational efficiency suitable for mesoscale modeling.^324–326^ MLIPs can function as high-fidelity reference potentials for bottom-up coarse-graining, thereby enabling CG models to inherit chemically specific interactions, such as hydrogen bonding, acid–base interactions, and interfacial charge regulation. These interactions have a significant impact on the dynamics that are investigated by NBS, NSE, and XPCS techniques.

Taken together, these advancements underscore how MLIPs, CGMD, XPCS/NSE/NBS, and ML-enabled scattering inversion are now firmly part of a shared, coevolving ecosystem for understanding PNCs.^285,319–323^ Simulation and scattering have long advanced in tandem; what is emerging today is a deeper and more seamless integration in which chemically specific atomistic models, mesoscale coarse-grained simulations, and data-driven scattering analyses operate on the same footing and increasingly inform one another. Foundation-scale MLIPs now provide the accuracy and transferability needed to generate chemically realistic training data for bottom-up coarse-grained models. Meanwhile, ML-assisted inversion of neutron and X-ray scattering enables quantitative access to anisotropy, relaxation spectra, and mesoscale structural descriptors that were previously difficult to extract. This expanding methodological interplay is accelerating our ability to interpret hierarchical interfacial dynamics, map mobility gradients across the interphase, and connect nanoscale chemistry to macroscopic reinforcement. As these tools continue to evolve and converge, the use of *scattering-guided simulation* and *simulation-guided scattering* will increasingly serve as a unified, iterative framework for designing, interrogating, and optimizing advanced PNCs.

## Abbreviations and Acronyms

AMD: All-Atom Molecular Dynamics
BDS: Broadband Dielectric Spectroscopy
BP: Bound Polymer
CB: Carbon Black
CGMD: Coarse-Grained Molecular Dynamics
dPB: Deuterated Polybutadiene
DSC: Differential Scanning Calorimetry
EFTEM: Energy Filtered Transmission Electron Microscopy
hPB: Hydrogenated Polybutadiene
IXS: Inelastic X-ray Scattering
MCT: Mode-Coupling Theory
ML: Machine-Learning
MLIPs: Machine-Learned Interatomic Potentials
MLS: Multiline frequency Mössbauer absorption spectrometer
MMA: Methyl Methacrylate
NBS: Neutron Backscattering
NMR: Nuclear Magnetic Resonance
NPA: Neutron Polarization Analysis
NPs: Nanoparticles
NSE: Neutron Spin Echo
PNCs: Polymer Nanocomposites
P2VP: Poly­(2-vinyl pyridine)
QEGS: Quasielastic γ-ray Spectroscopy
QENS: Quasielastic Neutron Scattering
*R*_g_: Radius of Polymer Gyration
SANS: Small-Angle Neutron Scattering
SAXS: Small-Angle X-ray Scattering
SCA: Silane Coupling Agent
SiO_2_: Silica
Si266: Bis­(3-triethoxysilylpropyl)­disulfide
TDI: Time Domain Interferometry
TEM: Transmission Electron Microscopy
*T*_g_: Glass Transition Temperature
TOF: Time-of-Flight Spectroscopy
USAXS: Ultra-Small-Angle X-ray Scattering
WASP: Wide Angle Spin Echo
WLF: Williams–Landel–Ferry
XPCS: X-ray Photon Correlation Spectroscopy
XR: X-ray Reflectivity
